# N-terminal region is responsible for mHv1 channel activity in MDSCs

**DOI:** 10.3389/fphar.2023.1265130

**Published:** 2023-10-17

**Authors:** Antonio Peña-Pichicoi, Miguel Fernández, Nieves Navarro-Quezada, Juan J. Alvear-Arias, Christian A. Carrillo, Emerson M. Carmona, Jose Garate, Angelica M. Lopez-Rodriguez, Alan Neely, Erick O. Hernández-Ochoa, Carlos González

**Affiliations:** ^1^ Centro Interdisciplinario de Neurociencia de Valparaíso, Universidad de Valparaíso, Valparaíso, Chile; ^2^ Millennium Nucleus in NanoBioPhysics, Universidad de Valparaíso, Valparaíso, Chile; ^3^ Texas Tech University Health Sciences Center, Lubbock, TX, United States; ^4^ Facultad de Ingeniería y Tecnología, Universidad San Sebastián, Santiago, Chile; ^5^ Facultad de Ciencias Químicas, Universidad Juárez del Estado de Durango, Durango, Mexico; ^6^ Department of Biochemistry and Molecular Biology, School of Medicine, University of Maryland, Baltimore, MD, United States; ^7^ Department of Physiology and Biophysics, Miller School of Medicine, University of Miami, Miami, FL, United States

**Keywords:** voltage-gated proton channel, cloning, mHv1.1, mHv1.2, mHv1.3, myeloid-derived suppressor cells, immunosuppression

## Abstract

Voltage-gated proton channels (Hv1) are important regulators of the immunosuppressive function of myeloid-derived suppressor cells (MDSCs) in mice and have been proposed as a potential therapeutic target to alleviate dysregulated immunosuppression in tumors. However, till date, there is a lack of evidence regarding the functioning of the Hvcn1 and reports on mHv1 isoform diversity in mice and MDSCs. A computational prediction has suggested that the Hvcn1 gene may express up to six transcript variants, three of which are translated into distinct N-terminal isoforms of mHv1: mHv1.1 (269 aa), mHv1.2 (269 + 42 aa), and mHv1.3 (269 + 4 aa). To validate this prediction, we used RT-PCR on total RNA extracted from MDSCs, and the presence of all six predicted mRNA variances was confirmed. Subsequently, the open-reading frames (ORFs) encoding for mHv1 isoforms were cloned and expressed in *Xenopus laevis* oocytes for proton current recording using a macro-patch voltage clamp. Our findings reveal that all three isoforms are mammalian mHv1 channels, with distinct differences in their activation properties. Specifically, the longest isoform, mHv1.2, displays a right-shifted conductance–voltage (GV) curve and slower opening kinetics, compared to the mid-length isoform, mHv1.3, and the shortest canonical isoform, mHv1.1. While mHv1.3 exhibits a V_0.5_ similar to that of mHv1.1, mHv1.3 demonstrates significantly slower activation kinetics than mHv1.1. These results suggest that isoform gating efficiency is inversely related to the length of the N-terminal end. To further explore this, we created the truncated mHv1.2 ΔN20 construct by removing the first 20 amino acids from the N-terminus of mHv1.2. This construct displayed intermediate activation properties, with a V_0.5_ value lying intermediate of mHv1.1 and mHv1.2, and activation kinetics that were faster than that of mHv1.2 but slower than that of mHv1.1. Overall, these findings indicate that alternative splicing of the N-terminal exon in mRNA transcripts encoding mHv1 isoforms is a regulatory mechanism for mHv1 function within MDSCs. While MDSCs have the capability to translate multiple Hv1 isoforms with varying gating properties, the *Hvcn1* gene promotes the dominant expression of mHv1.1, which exhibits the most efficient gating among all mHv1 isoforms.

## 1 Introduction

The mHv1 proton channel mediates the movement of protons across cell membranes, supporting diverse vital cellular processes. These processes include intracellular pH regulation, apoptosis, proliferation, and migration and are well-documented in the immune system, such as in B and T lymphocytes (Hondares et al., 2014; Sasaki, M., et al., 2013; Asuaje et al., 2017; Coe, D., et al., 2022). mHv1 channel dysregulation leads to various immune disorders, such as autoimmune disorders (Sasaki, M., et al., 2013) or T-cell maturation problems (Coe, D., et al., 2022). Recently, the functional expression of the voltage-gated Hv1 channel was reported in myeloid-derived suppressor cells (MDSCs) (Alvear-Arias, J., et al., 2022), a population of immune cells characterized by strong T-lymphocyte suppressor activity (Gabrilovich, D.I., 2017). In MDSCs, the mHv1 function supports the immunosuppressive activity (Alvear-Arias, J.J., et al., 2022). This discovery increases the potential therapeutic value of Hv1, as cancers are known to recruit the MDSC to its microenvironment (Kusmartsev, S., et al., 2008; Corzo, C. A., et al., 2009; Yan, L., et al., 2014; Alvear-Arias, J.J., et al., 2022; Cozzolino, M., et al., 2023; Qin, G., et al., 2023).

While genetic and pharmacological approaches hold promise for restoring homeostasis in MDSC immunosuppression through mHv1, our current understanding of the Hv1 gene in mice and its function remains limited. Additionally, there is a lack of comprehensive characterization of the *Hvcn1* gene and also of empirical evidence of mHv1 isoform diversity in mice. These factors currently hinder the realization of mHv1 as a therapeutic target for MDSCs. However, it is intriguing to note that computational predictions regarding the *Hvcn1* gene suggest the possibility of alternative splicing, potentially resulting in the expression of three isoforms with varying N-terminal lengths in mice.

In our study, we first validated the predicted alternative expression of *Hvcn1* gene isoforms by conducting reverse transcription polymerase chain reaction (RT-PCR) on total RNA extracted from MDSCs. This validation confirmed the presence of all six predicted *Hvcn1* transcripts. Then, we successfully cloned three open-reading frames (ORFs) associated with potential mHv1 isoforms. Furthermore, we observed a nonlinear voltage-dependent behavior of currents, characteristic of ion channels, in all cloned ORFs using a macro-patch voltage-clamp. Next, we biophysically characterized these channels using inside-out macro-patches and observed that all cloned ORFs functioned as *bona fide*, mammalian mHv1 channels. Since these mHv1 isoforms possess a different amino-terminus, we were intrigued by the potential differences in their functionality. When focusing on their activation process, we noticed significant differences among the isoforms. The longest isoform, mHv1.2, exhibited a V_0.5_ shift of nearly 1 kT (25 mV) toward depolarized potentials and slower opening kinetics compared to the mid-length isoform, mHv1.3. Interestingly, the mHv1.3 isoform displayed no differences in its V_0.5_ when compared to mHv1.1, the canonical and shortest isoform, but exhibited slower activation kinetics than mHv1.1. Based on these findings, we propose that the different isoforms display differences in their activation. Specifically, mHv1.1 exhibits the most “efficient” gating, followed by mHv1.3, and lastly, mHv1.2, which shows less efficient activation. In this regard, we will use the term gating “efficiency” to denote an increased probability of the channel being open at a given voltage range, which includes activating in a more negative voltage interval and/or opening more rapidly. Interestingly, the only reported Hv1 isoform across species is found in humans, known as HVCN1S, a shortened isoform of the canonical HVCN1L, which lacks the initial 20 amino acids and exhibits slightly slowed activation. Given that the isoforms reported here differ in their N-terminal length due to alternative splicing at the initial exons, we investigated whether the length of the amino-terminus regulates the gating of mHv1 isoforms. To explore this, we engineered a truncated form of the longest mHv1.2 isoform lacking the initial 20 amino acids, designated as mHv1.2 ΔN20. We predicted and confirmed a gating phenotype intermediate between the longest isoform, mHv1.2, and the shortest isoform, mHv1.1. The conductance–voltage (GV) curve was shifted to the left compared to mHv1.2 and to the right compared to mHv1.1. These differences were consistently observed at the single-channel level, and the opening kinetics was faster than that of mHv1.2 but slower than that of mHv1.1. In conclusion, our findings provide valuable insights into the genetics of *Hvcn1* and the diversity of mHv1 isoforms in immune cells of mice.

These results were discovered in mouse MDSCs, where the *Hvcn1* gene alternatively splices at the initial exons of mRNAs encoding mHv1, thereby regulating the extension of their ORFs at the very beginning. Consequently, *Hvcn1* can express three mHv1 isoforms with different activation properties, determined by their N-terminal length, which in turn regulates their gating. Redundant mRNA expression of *Hvcn1* in MDSCs favors the expression of the shortest mHv1 isoform, which demonstrates a more efficient gating. It is possible for these longer isoforms to be still expressed, presumably depending on the genetic/phenotypic state of the cell.

## 2 Results

### 2.1 Cloning and function of *Hvcn1* gene transcripts encoding mHv1 isoforms from MDSCs

**FIGURE 1:**
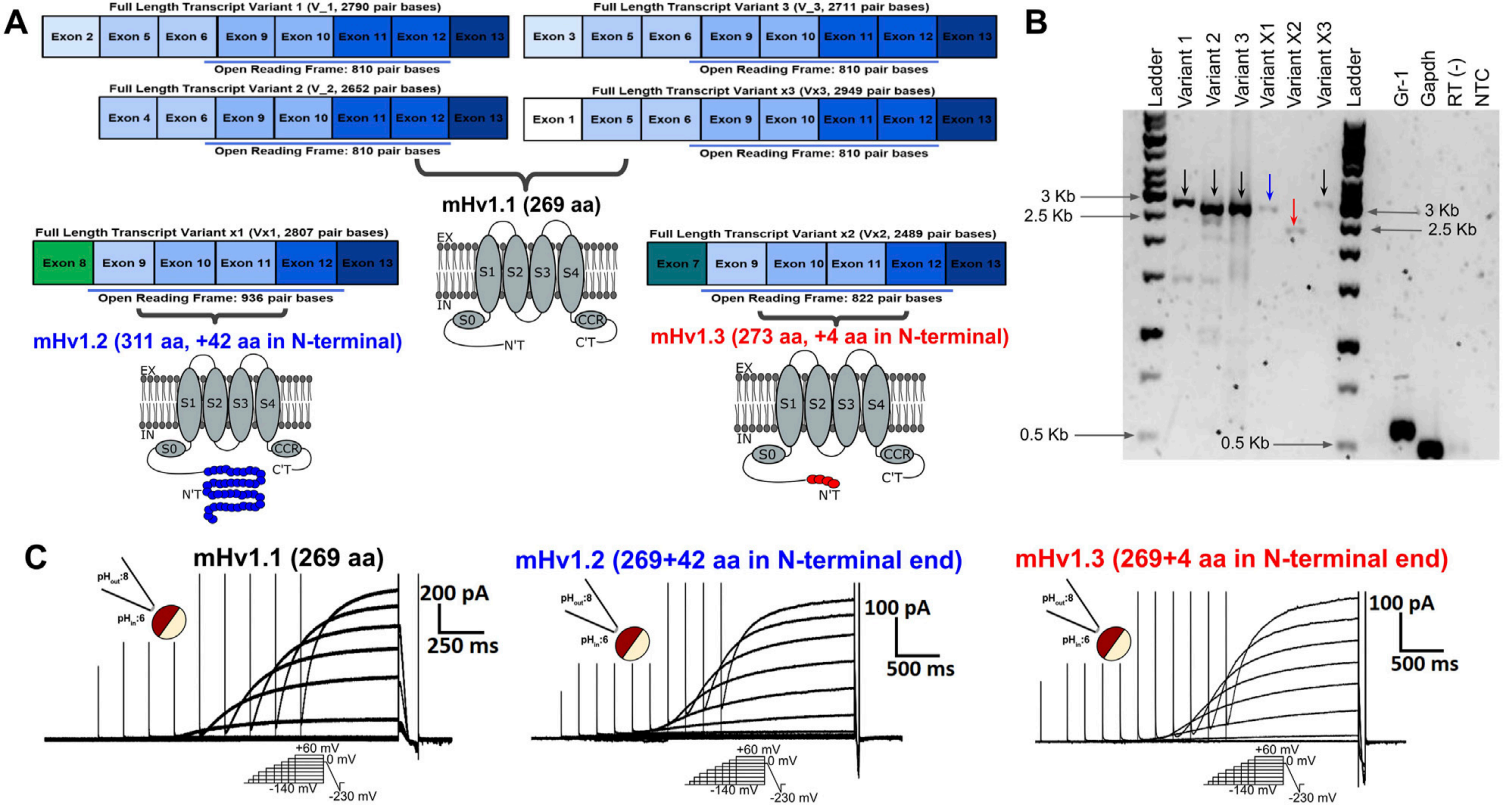
Predicted alternative splicing for the Hvcn1 gene could lead to the expression of transcripts exhibiting voltage-dependent function. **(A)** Images of rectangular boxes represent the length and exonic composition of the six transcript variants predicted to be expressed by the voltage-gated proton channel gene in mice, Hvcn1. Their ORF length is highlighted with a light blue bar. All the exons from 9 to 13 are predicted to be conserved among all the Hvcn1 transcripts. Transcript variants 1, 2, and 3 were validated and reported *in vitro*, while transcript variants x, x1, and x2 are still predicted. From these transcripts, it is predicted to express mHv1.1 (black), mHv1.2 (blue), and mHv1.3 (red) isoforms at a membrane, pointing out the additional amino acids each isoform could have in comparison with the canonical mHv1.1. They show their main domains, such as the transmembrane segments (S) 0 to 4, the intracellular arranged coiled-coil region (CCR), the C-terminal domain (C’T), and the N-terminal domain (N’T), that focus the distinctive amino acidic differences among these isoforms. **(B)** MDSC Hvcn1 transcripts were amplified by RT-PCR. All predicted variants were expressed besides the reported variants 1–3. The marker for Gr1 was used as the MDSC control. GAPDH was used as the positive control, while RT (−) (amplification without the retrotranscriptase enzyme) and NTC (non-template control) were used as negative controls. Arrow colors are related to the color symbology used for mHv1 isoforms in **(A)**. **(C)** Voltage-dependent outward currents from the different ORFs cloned from transcripts expressed from Hvcn1. Representative family of currents from oocytes expressing mHv1.1 (left, black), mHv1.2 (middle, blue), and mHv1.3 (right, red). Upper left inset: image representation of a macro-patch in inside-out configuration at ΔpH = 2. Bottom inset: voltage protocol applied during the experiment. Upper right inset: scale bar of intensity versus time.

Computational analysis of nucleotide sequences encoding transcript variants of the *Mus musculus Hvcn1* gene, located on Chromosome 5–[NC_000071.6 (122206805-122242297)], predicts the presence of up to 13 exons (Table 1). These exons have the potential for alternative splicing, resulting in the coding of at least six Hv proton channel mRNA transcripts (Figure 1A, left). Previous *in vitro* studies have functionally confirmed three transcripts coding for the same 269-amino acid mHv1.1 protein: variants 1 (NM_001042489.2), 2 (NM_028752.3), and 3 (NM_001359454.1). Notably, there is still a predicted isoform (variant X3: XM_006530472.3) encoding the same amino acid sequence, suggesting that the expression of the mHv1.1 isoform plays a significant role, possibly related to its translational redundancy among different mRNA transcripts.

**TABLE 1:** Exonic arrangement of the Hvcn1 gene. The genomic sequence NC_000071.6:122206805-122242297 from the *Mus musculus* strain C57BL/6J chromosome 5, GRCm38.p6, is predicted to be the Hvcn1 gene, with an extension of 35,493 pair bases. Length reported in pair bases. Sequence’s start and end reported in base pairs in direction 5′ to 3′.

Exon number	Length	Sequence start	Sequence end	Type
1	352	122344868	122345219	<exon>GT
2	193	122347792	122347984	<exon>GT
3	114	122348202	122348315	<exon>GT
4	106	122348350	122348455	<exon>GT
5	51	122348405	122348455	AG<exon>GT
6	132	122354295	122354426	AG<exon>GT
7	75	122358247	122358321	<exon>GT
8	393	122370356	122370748	<exon>GT
9	273	122371483	122371755	AG<exon>GT
10	105	122375744	122375848	AG<exon>GT
11	232	122376469	122376700	AG<exon>GT
12	113	122378277	122378389	AG<exon>GT
13	1,691	122378670	122380360	AG<exon>

The selective splicing of mHv1 channel isoforms can be explained by considering alternative transcription mechanisms. Within the six mRNA transcripts of the *Hvcn1* gene, four (transcript variants 1, 2, 3, and X3) begin processing at exons 2, 4, 3, and 1, respectively. The untranslated regions (UTRs) are located in these exons. Although the initiation codon for protein translation is found in exon 6, all of these transcripts encode the same 810-base pair (bp) product up to exon 13 (Figure 1A, left; Supplementary Figures S1A–C). This product corresponds to the canonical mHv1.1 isoform, consisting of 273 amino acids (Figure 1A, right).

For Vx1 (XM_011248243.2), transcription processing begins at exon 8, where the start codon for translation is also located. This results in an elongated ORF that codes for a 936-bp product (Figure 1A, left; Supplementary Figure S1B). This product corresponds to a predicted mHv1.2 isoform of 311 amino acids (Figure 1A, right).

Similarly, for Vx2 (XM_006530471.4), the transcription processing occurs at exon 7, where the transcription initiation site is located. This produces an mRNA with an ORF of 822 pb (Figure 1A, left; Supplementary Figure S1C). This allows the translation of the putative mHv1.3 channel isoform, which is only four amino acids longer than the canonical mHv1.1 (Figure 1A, right).

After the N-terminal variable region, all of the ORFs include the same coding sequence from exons 9 to 13. This results in all of the amino acid differences among mHv1 isoforms being concentrated in the initial N-terminal domain (Figure 1A, right; Supplementary Figure S1).

A comprehensive analysis of the primary sequences of the predicted longer isoforms revealed a high degree of conservation in the typical structural features for functional Hv1 proton channels (Supplementary Figure S1D), which include the selectivity filter at the residue D108 (Musset et al., 2011; DeCoursey et al., 2016); the voltage sensor domain (VSD), encompassing the S4 helix and its three arginines (referred to as R1 to R3, from outside to inside of the cell), which is the sensor of both voltage and ΔpH (Gonzalez et al., 2010; Gonzalez et al., 2013; Schladt and Berger, 2020; Carmona et al., 2021); and the coiled-coil region that promotes dimerization between Hv1 monomers (Koch et al., 2008; Gonzalez et al., 2010; Fujiwara et al., 2012; Fujiwara et al., 2012).

In the earlier prediction, it was mentioned that the *Hvcn1* gene has the potential for alternative splicing, which could result in the production of up to six mRNA transcript variants. These variants, in turn, translate into three isoforms of the mHv1 channel protein with different N-terminal region lengths: mHv1.1, mHv1.2, and mHv1.3.

To further investigate this, we designed specific primers targeting the full length of the six predicted transcript variants, including the 5′ and 3′UTRs. We then screened a messenger library derived from mouse MDSCs using RT-PCR with these primers (Table 2).

**TABLE 2:** Primers used for Hvcn1 transcript identification.

Sense	Primer (5′ to 3′)	Tm (°C)	Restriction enzyme site
Forward Hvcn1 variant 1	GTT​ACC​ATG​GTC​CGT​AGG​CCT​TTG​AGT​TTG​CAG	75	NcoI
Forward Hvcn1 variant 2	GTT​ACC​ATG​GGC​CAC​ACC​CAG​CAT​CTT​GTG​A	77	NcoI
Forward Hvcn1 variant 3	ATT​ACC​ATG​GCA​CCG​TGG​GGT​GAC​CAA	77	NcoI
Forward Hvcn1 variant X1	AGA​TCC​ATG​GCT​GTC​TGT​TGT​GTG​ACC​ATG​TG	75	NcoI
Forward Hvcn1 variant X2	GTT​ACC​ATG​GAA​GGT​CTT​GAG​GCC​TGG​AAC​A	75	NcoI
Forward Hvcn1 variant X3	GAT​TCC​ATG​GTG​CTC​TGT​CAT​TTG​GTC​CTT​TTA​TGT​CC	74	NcoI
Reverse Hvcn1 variants	AAA​AGA​CGT​CAC​AGA​AAG​CCC​CAT​GAC​AGC​AA	75	AatII
Forward Gr1	ATT​GTA​TTG​GGG​TCC​CAC​CTG​AGA	70	—
Reverse Gr1	CAC​ATG​CCT​CCA​GGG​TCA​AGA	70	—
Forward Cd11b	CTC​TCA​AGA​AGA​ATG​TCC​TCA​GCA	66	—
Reverse Cd11b	ACG​TGT​TCA​CCA​GCT​GGC​TTA	69	—
Forward GAPDH	CAA​GGT​CAT​CCA​TGA​CAA​CTT​TG	64	—
Reverse GAPDH	GTC​CAC​CAC​CCT​GTT​GCT​GTA​G	70	—
Forward Hvcn1 variant 2 ORF	GTT​AGA​GCT​CAA​CAG​GAA​AAT​GAC​TTC​CCA​TGA​CC	73	SacI
Forward Hvcn1 variant x1 ORF	GTT​AGA​GCT​CCC​GTA​TCC​AAG​AAT​GGA​CA	71	SacI
Forward Hvcn1 variant x2 ORF	GTT​AGA​GCT​CAC​AGG​CTG​AAT​GGA​AAC​AGA​ATG​GC	75	SacI
Reverse Hvcn1 variants ORF	TTA​TGG​ATC​CCG​GAG​TGT​CTG​GAA​GGC​TAG​TT	75	BamHI

The RT-PCR analysis results revealed the presence of four bands matching the expected sizes for variants 1–3 and variant X3 mRNAs, which encode the mHv1.1 isoform (2,790 pb for V_1, 2,652 pb for V_2, 2,711 pb for V_3, and 2,949 pb for Vx3, Figure 1B, indicated by black arrows). Another band of the expected size was detected for the transcript variant X1, which expresses mHv1.2 (2,807 pb for Vx1, Figure 1B, indicated by a blue arrow). Finally, a band corresponding to the transcript variant X2 was observed, which expresses the mHv1.3 isoform (2,489 pb for Vx3, Figure 1B, indicated by a red arrow).

To confirm the identity of these amplified DNAs, they were sequenced and determined to be mRNA transcripts derived from the *Hvcn1* gene. Based on this confirmation, the ORFs that encode hypothetical mHv1.1, mHv1.2, and mHv1.3 proton channels were cloned.

To assess the functionality of these hypothetical ion channels, we conducted electrophysiological recordings in *Xenopus laevis* oocytes, which does not express endogenous proton currents (Supplementary Figures 1E,F), and injected them with mHv1.1, mHv1.2, and mHv1.3 cRNA. As predicted, these sequences should contain all the structural components of functional mHv1 channels (Supplementary Figure S1D). By using the voltage patch-clamp technique, we tested their hypothetical function as voltage-sensitive channels using excised macro-patches in the inside-out configuration. We recorded voltage-activated outward currents for mHv1.1, mHv1.2, and mHv1.3 (Figure 1C, left to right, respectively. Methods; Eq. 1).

### 2.2 Biophysical characteristics of cloned transcripts are consistent with those of mammalian mHv1 isoforms

While the products expressed by the *Hvcn1* gene in *Xenopus* oocytes exhibited ion channel behavior, the question remains: Do these channels function as mammalian voltage-gated proton channels? To address this question, we investigated whether the cloned ORFs exhibit the hallmarks of mHv1 channels. In addition to voltage-dependent gating (Sasaki, M., Takagi, M., & Okamura, Y., 2006; Ramsey, I. S., et al., 2006), these include ΔpH-dependent gating (Musset, B., et al., 2008; Schladt, T. M., & Berger, T. K., 2020; Carmona, E. M., et al., 2021), unitary currents in the order of femtoamperes (fA) (Cherny, A., et al., 2003), proton selectivity (Musset, B., et al., 2011), and a dimeric oligomer state (Lee, S. Y., Letts, J. A., & MacKinnon, R., 2008; Koch, H. P., et al., 2008; Gonzalez, C., et al., 2010; Fujiwara, Y., et al., 2012; Gonzalez et al., 2013) in the cloned ORFs.

**FIGURE 2:**
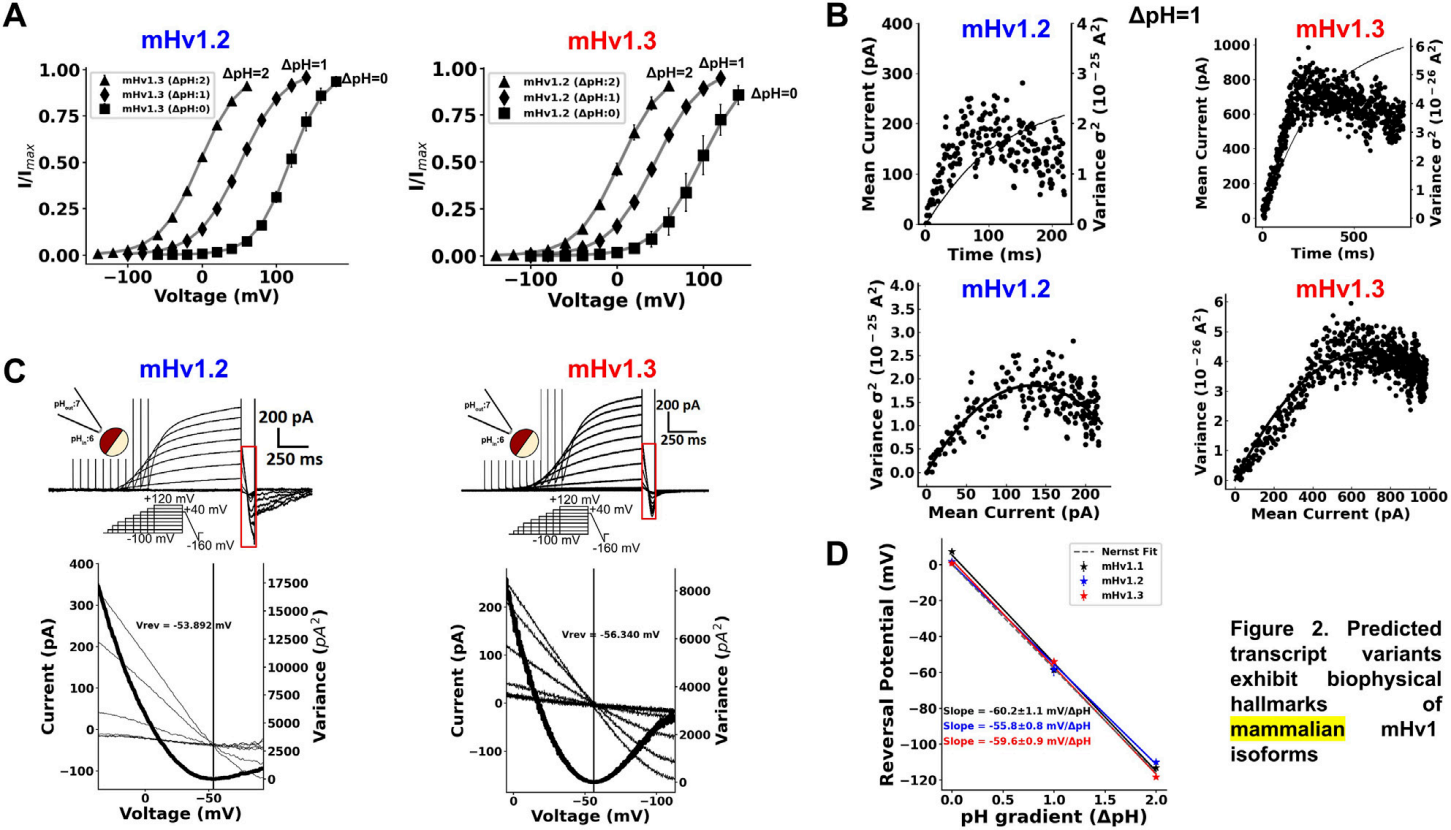
Predicted transcript variants exhibit biophysical hallmarks of mammalian mHv1 isoforms. **(A)** ∆pH dependence of IV curves for mHv1.2 (blue) and mHv1.3 (red) currents, respectively. The parameters of the fit of the data to the normalized Boltzmann function in Eq. 2, V_0.5_, and zδ are tabulated in Table 3. **(B)** Representative results from nonstationary noise analysis at ΔpH = 1 (pHin:6; pHex:7) of mHv1.2 (blue, left) and mHv1.3 (red, right) at 100 mV and 80 mV, respectively. (top) Time course of the mean current and the variance of mHv1.2 and mHv1.3, whose noise exhibits a biphasic behavior, characteristic of the probabilistic opening process of ion channels, with the first part pointing to a variance maximum, which corresponds to the point where half of the channels in the ensemble are open, and the second part pointing to the effective maximum open probability of the isoforms (bottom). Variance versus mean current plots for mHv1.2 and mHv1.3, with both data showing a parabolic behavior. These data were fitted to the parabolic Eq. 3, obtaining microscopical parameters of mHv1.2 and mHv1.3, such as the number of channels, with 9,477 mHv1.2 channels and 106,900 mHv1.3 channels; the unitary current, with 28 fA for mHv1.2 and 12.7 fA for mHv1.3; the maximum open probability, with 0.82 for mHv1.2 and 0.73 for mHv1.3; and the unitary conductance, with 177 fS for mHv1.2 and 91.9 fS for mHv1.3. **(C)** (upper) Representative currents for mHv1.2 (left, blue) and mHv1.3 (right, red) at ∆pH1 and with a depolarizing shortened protocol that avoids depletion (middle inset). The red rectangle is delimiting the fast ramp that is used to estimate the reversal potential (bottom). Magnification of the delimited red rectangle, showing the crossing of the proton currents and the minimum variance (bold curve) achieved where the currents are reversed (vertical black straight line) for mHv1.2 (left, blue) and mHv1.3 (right, red). **(D)** Reversal potentials plotted against pH gradients (∆pH) for mHv1.1, mHv1.2, and mHv1.3. Circles represent the experimental data; the black, blue, and red straight lines symbolize the fit to the data; and the dotted gray straight line represents the prediction of the Nernst equation of the equilibrium of protons.

All ORF currents exhibited a clear ΔpH dependence (Methods, Eq. 2), where the IV curves in all isoforms shifted to hyperpolarized potentials with increase in the ΔpH and toward depolarized potentials when the ΔpH was lower, similar to mammalian Hv1 channels (Figure 2A; Supplementary Figure S2A; Table 3; Musset, B., et al., 2008). These results suggest that the gating of the cloned ORFs is ΔpH-dependent.

**TABLE 3:** Parameters of fits of IVs with a Boltzmann function for different gradients of pH.

Isoform	ΔpH (pHi/pHo)	zδ	V_0.5_ (mV)	n
	0 (7/7)	1.49 ± 0.40	98.52 ± 13.85	3
Hv1.1	1 (7/6)	1.30 ± 0.24	43.38 ± 13.24	3
	2 (8/6)	1.15 ± 0.09	−16.89 ± 4.23	5
	0 (7/7)	1.04 ± 0.003	96.57 ± 10.79	3
Hv1.2	1 (7/6)	0.96 ± 0.02	44.35 ± 1.85	3
	2 (8/6)	1.03 ± 0.06	4.24 ± 3.59	3
	0 (7/7)	1.10 ± 0.10	118.77 ± 4.35	3
Hv1.3	1 (7/6)	0.89 ± 0.01	51.98 ± 3.54	3
	2 (8/6)	0.94 ± 0.04	−2.88 ± 0.13	3

The unitary conductance of mammalian Hv1 channels is estimated to be in the order of fS (Cherny, A., et al., 2003), with single-channel current values below the limit of detection of traditional electrophysiology techniques, including inside-out macro-patches. Consequently, direct single-channel current measurements are not feasible.

Using nonstationary noise analysis (Alvarez, O., Gonzalez, C., & Latorre, R., 2002), we determined the unitary conductance from the macroscopic currents of the three cloned ORFs. In Figure 2B (top), the time course for the mean current and variance for mHv1.2 and mHv1.3 is shown, both obtained at ΔpH = 1 (pHin = 6 and pHout = 7) at 100 mV and 80 mV, respectively. Both variances exhibited a biphasic time course, reflecting the open probability of the channels (Gonzalez, et al., 2000). From these data, variance versus mean current plots were generated (Figure 2B, bottom), which were fitted to Eq. 3 (see *Methods*). The unitary conductance values obtained for mHv1.2 and mHv1.3 were 115.1 ± 34.8 fS (*n* = 3) and 164.4 ± 24.8 fS (*n* = 4), respectively. Additionally, the maximum open probabilities for mHv1.2 and mHv1.3 were 0.86 ± 0.03 (*n* = 3) and 0.80 ± 0.03 (*n* = 4), respectively.

In Supplementary Figures S2B, nonstationary noise analysis for mHv1.1, mHv1.2, and mHv1.3 at ΔpH = 2 (pHin = 6 and pHout = 8) and at 0 mV is displayed. The fits to the variance versus mean current data exhibited unitary conductance values of 93.6 ± 16.9 fS (*n* = 4) for mHv1.1, 63.1 fS for mHv1.2 (*n* = 1), and 165.4 ± 79 fS for mHv1.3 (*n* = 3), with a maximum open probability of 0.85 ± 0.04 (*n* = 4) for mHv1.1, 0.99 for mHv1.2, and 0.89 ± 0.08 (*n* = 3) for mHv1.3. All these data are consistent with previously reported unitary properties for Hv1 channels (Cherny et al., 2003).

To confirm the proton selectivity of all cloned isoforms, we employed variable duration voltage protocols coupled to a fast ramp ranging from depolarizing to hyperpolarizing potentials (Figure 2C), as previously utilized (Gonzalez, C., et al., 2010; Carmona et al., 2021; Alvear-Arias, J., Carrillo, C., et al., 2022). Using this protocol, we measured mHv1.2 and mHv1.3 currents at ΔpH = 1 (Figure 2C, upper panel). The red boxed region highlights the current during the fast ramp (Figure 2C). The lower panels in Figure 2C provide a closer examination of the currents elicited by the fast repolarization ramp, revealing an overlap of the currents for all the cloned channels. To determine the exact voltage at which the current reverses, we calculated the variance of the current over time and focused on the point where this variance was minimal (Figure 2C, lower panel, red straight line, Methods, Eqs 4–10). This allowed us to estimate reversal potential values near the Nernst equilibrium for protons at ΔpH = 1 (Figure 2C, lower panel). We repeated the measurements at ΔpH = 2 and ΔpH = 0 (Supplementary Figure S2C), and the estimated reversal potentials, along with those previously obtained at ΔpH = 1, were plotted against ΔpH (Figure 2D). Every cloned channel exhibited a straight-line slope between −55.8 mV and 60.2 mV/ΔpH (Figure 2D). As the Nernst equilibrium for protons predicts a slope of −58 mV/ΔpH, our data strongly suggest that currents from mHv1 isoforms are selective for protons.

The previous information we have collected is consistent with the idea that all cloned ORFs are functional voltage-gated proton channel isoforms**.** In mammals, Hv1 is naturally found as a dimer (Fujiwara, Y., et al., 2012). The novel cloned mHv1 isoforms contain a predicted C-terminal domain that is expected to fold into a coiled-coil region (CCR) (Figure 1A, right), driving the dimerization process. For all isoforms, we observed a sigmoidal pattern on activation currents (Figure 1C), a characteristic that is consistent with the strong cooperativity previously reported for dimeric Hv1 formation (Gonzalez et al., 2010).

Next, to determine the oligomeric state of the cloned Hv1 isoforms, we applied the limiting slope technique method at very low open probabilities to estimate the effective gating charge (zδ_eff_) coupled to the channel opening (Gonzalez, C., et al., 2013). The three arginines in the S4 helix contribute directly to the value of the zδ_eff_ in Hv1 channels (Gonzalez, C., et al., 2013), with a monomer yielding a value of ∼3 charges and a Hv1 dimer reaching a value of ∼6 charges (Gonzalez, C., et al., 2010; Gonzalez, C., et al., 2013). In mice, limiting slope measurements of Hv1 indicated charge values of 4 e_0_ for dimers and 2 e_0_ for monomers (Fujiwara, Y., et al., 2012).

We used slow voltage ramps (ranging between 0.5 mV/s and 1 mV/s) to ensure gating equilibrium during measurements of mHv1.1, mHv1.2, and mHv1.3 currents, which were then transformed into macroscopic conductance and graphically represented in a log-linear plot against voltage (Supplementary Figure S3A). This analysis followed the limiting slope theory for Hv1, as discussed elsewhere (Gonzalez et al., 2010; Gonzalez et al., 2013; Methods, Eqs 11, 12). We used a Boltzmann curve to fit the previous data and estimated a zδ_eff_ of 4.19 ± 0.13 e_0_, 4.30 ± 0.18 e_0_, and 4.44 ± 0.17 e_0_ for mHv1.1, mHv1.2, and mHv1.3, respectively (Supplementary Figure S3B). These values are consistent with all these novel isoforms of mHv1 having a dimeric oligomerization, as observed in mammalian mHv1 channels.

All the previous gathered evidence suggests that the three cloned ORFs from *Hvcn1* gene expression in MDSCs are indeed three mammalian isoforms of mHv1 channels: mHv1.1 (the shortest), mHv1.2 (the longest), and mHv1.3 (of mid-length). Although these isoforms exhibit clear hallmarks of mammalian mHv1 channels, do they exhibit functional differences among them?

### 2.3 Isoforms of mHv1 exhibit differences in their voltage-dependent activation

**FIGURE 3:**
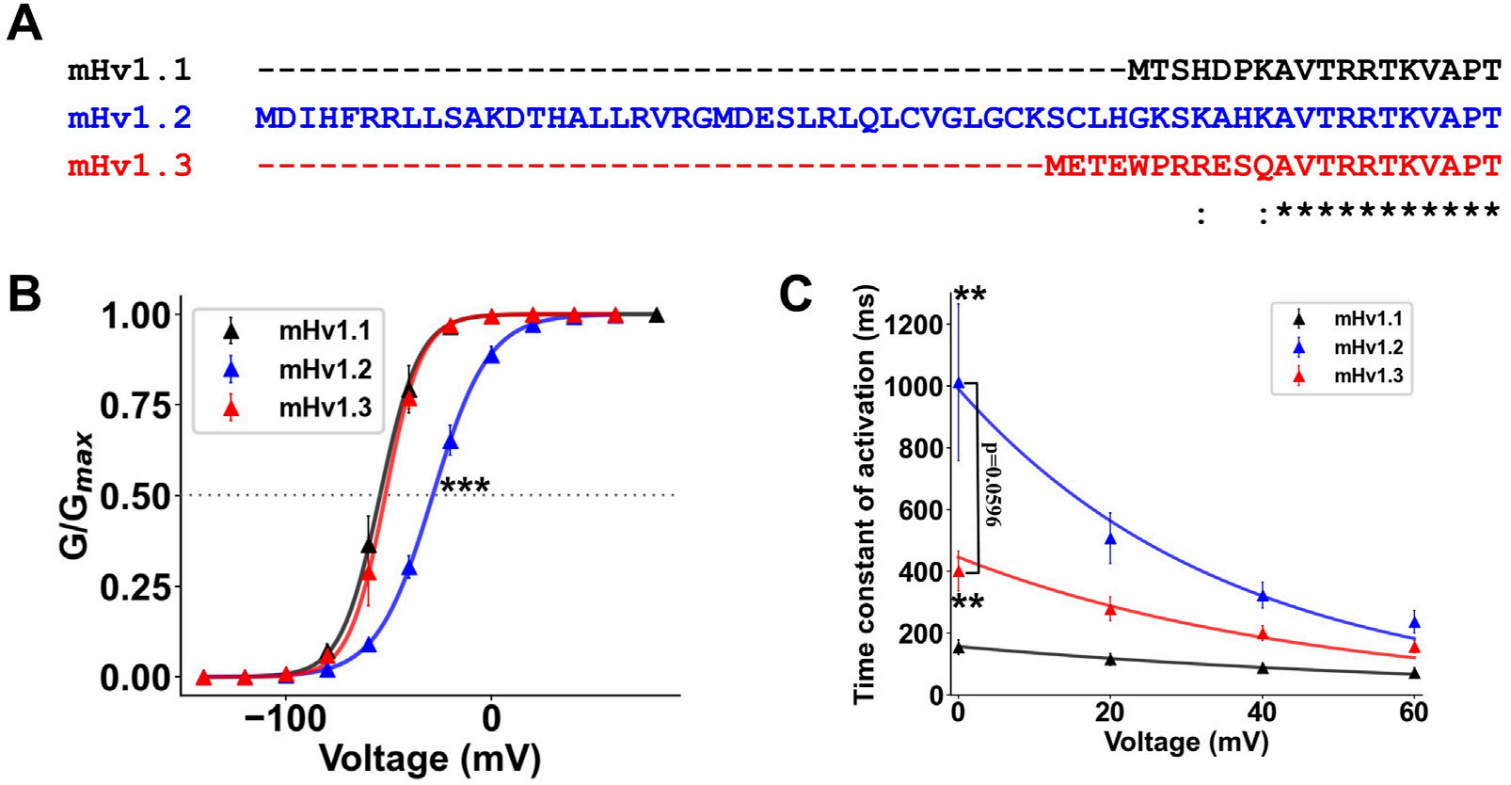
The longer isoforms of mHv1 possess a differential gating of their function. **(A)** Multiple alignment between the predicted primary sequences of the identified mHv1 isoforms, showing amino acid differences at the beginning of the N terminus. **(B)** Normalized conductance values at ΔpH = 2 (pHin:6; pHex:8) for isoforms of mHv1 (mHv1.1 in black, mHv1.2 in blue, and mHv1.3 in red) computed as G (V) = I/(V-Vrev). The data are expressed as mean ± SEM (*n* = 3) and fitted to a Boltzmann function (continuous line), G (V) = Gmax/1+exp [-zδF (V-V_0.5_)/RT] (Student’s t-test; ***p < 0.005). **(C)** Time constants plotted against voltage for isoforms of mHv1 obtained from macroscopic Hv1 currents. The values are shown as mean ± SEM (*n* = 3) (Student’s t-test; **p < 0.01 for mHv1.1 vs. mHv1.2 and mHv1.1 vs. mHv1.3; mHv1.2 vs. mHv1.3 exhibited *p* < 0.6).

The *Hvcn1* gene in mice expresses up to six mRNA transcripts, from which three ORFs are identified, expressing three different mHv1 isoforms. Upon inspecting their primary sequences, one can observe that nearly all sequences are conserved. However, at the very beginning of the N-terminal domain, there are 42 additional amino acids in mHv1.2 compared to mHv1.1 (Figure 3A) and four additional amino acids in mHv1.3 compared to mHv1.1 (Figure 3A). Could these differences in amino acids result in functional differences among mHv1 isoforms?

The sole reported isoform of Hv1, HVCN1_S_, has 20 fewer amino acids than the canonical Hv1, HVCN1_L_, particularly at the beginning of the N terminus (Hondares, et al., 2014). HVCN1_S_ displays slightly slower activation kinetics when compared to HVCN1_L_. Given the evident and localized differences in the beginning of the N-terminal domain of mHv1 isoforms, we hypothesize that each mHv1 isoform might possess distinctions in the gating properties. To address this hypothesis, we conducted a comprehensive study of the activation of mHv1 isoforms, centering on the functioning at the thermodynamic level (conductance/voltage relationship) and at the kinetic level (activation kinetics).

First, we calculated and fitted conductance vs. voltage curves for all three isoforms (Methods; Eqs 13, 14) and compared them at ΔpH = 2 (Figure 3B). We did not observe any difference between the V_0.5_ of mHv1.1 and the V_0.5_ of mHv1.3 in the GV plots (Student’s t-test; *p* = 0.68; Figure 3B; Table 4), with a clear rightward shift in the GV curve for mHv1.2 (Figure 3B; Table 4). The rightward shift in the GV curve of mHv1.2 is consistent with nearly a 1-kT displacement of the V_0.5_ to the right when compared to mHv1.1, and this difference is statistically significant (Student’s t-test; ***p < 0.005; Figure 3B; Table 4). This suggests that the 42 extra amino acids in mHv1.2 could stabilize the channel’s closure and/or destabilize the channel’s opening. On the other hand, the presence of four extra amino acids in mHv1.3 does not contribute to channel closure or destabilize the opening.

**TABLE 4:** Parameters of fits of GVs estimated at the tail with a Boltzmann function for different gradients of pH.

Isoform	ΔpH (pHi/pHo)	zδ	V_0.5_ (mV)	n
	0 (7/7)	1.44 ± 0.25	65.17 ± 0.18	3
Hv1.1	1 (7/6)	1.66 ± 0.56	−5.23 ± 0.32	3
	2 (8/6)	2.67 ± 0.13	−54.06 ± 3.57	4
	0 (7/7)	0.86 ± 0.09	83.52 ± 0.31	3
Hv1.2	1 (7/6)	2.10 ± 0.03	−0.77 ± 0.01	3
	2 (8/6)	1.88 ± 0.08	−28.54 ± 2.17	3
	0 (7/7)	1.11 ± 0.12	71.36 ± 0.44	3
Hv1.3	1 (7/6)	2.08 ± 0.23	5.86 ± 0.11	3
	2 (8/6)	2.88 ± 0.51	−51.89 ± 3.05	3

Next, we investigated the activation kinetics of these isoforms. We determined voltage-dependent time constants, which were then plotted against voltage (τ-V curves, Figure 3C; Methods; Eqs 15–17). It can be observed that mHv1.1 exhibits the fastest τ-V, followed by mHv1.3, and finally, the slowest, mHv1.2 (Figure 3C). At 0 mV, the time constants are statistically different (data were analyzed using Student’s t-test; **p < 0.01 for mHv1.1 vs. mHv1.2, **p = 0.01 for mHv1.1 vs. mHv1.3, and *p* = 0.06 for mHv1.2 vs. mHv1.3). These kinetics findings suggest that a longer N-terminal end leads to slower activation of mHv1.

Are these cloned mHv1 channels responsible for the proton currents seen in native murine tissues? The validation of cloned *Hvcn1* transcripts expressing functional mHv1 isoforms could support that mHv1 currents in MDSCs are produced by the combination of mHv1 isoforms. Previous studies have reported that Hv1 heterologous expression in HEK-293 or COS-7 cells shifts their V_0.5_ toward hyperpolarized potentials, reaching values near −30 mV when compared to mHv1 recorded in a system where it is expressed natively (Musset B., et al., 2008). To confirm that *X. laevis* oocytes are a suitable model for mimicking native mHv1 expression in MDSCs, we measured mHv1 currents from MDSCs by whole-cell patch-clamp and computed their normalized conductance as a function of voltage (Supplementary Figures S4A, B). We observed that the GV curve of mHv1 in MDSCs closely resembles the GV curves found for mHv1 isoforms expressed in *X. laevis* oocytes (Supplementary Figure S4B; Table 5). This suggests that mHv1 isoforms expressed in oocytes from *X. laevis* can effectively replicate the function observed in native mHv1 in MDSCs.

**TABLE 5:** Parameters of fits of GVs from ON macroscopic currents with a Boltzmann function at ΔpH = 2.

Hv1 type	V_0.5_ (mV)	zδ	n
mHv1.1	−50.97 ± 1.17	3.05 ± 0.44	3
mHv1.2	−25.19 ± 0.38	1.08 ± 0.17	3
mHv1.3	−44.23 ± 0.14	1.73 ± 0.10	3
Hv1 MDSC	−30.02 ± 0.78	1.63 ± 0.14	3

Subsequently, we investigated whether the mHv1 function in MDSCs is a result of the combination of mHv1 isoforms. We noted that all three isoforms exhibited different τ_activation_ at 0 mV (Figure 3C) and assumed that if all three mHv1 isoforms are expressed, fitting the mHv1 MDSC currents at 0 mV to the sum of three simple exponential functions (Methods, Eq. 18) should allow the estimation of three time constants, each related to a single isoform expressed by the *Hvcn1* gene. By performing this fit to mHv1 currents from MDSCs at 0 mV (Supplementary Figure S4C, inset), we obtained three ? and compared them to the τ_activation_ at 0 mV for all mHv1 isoforms (Supplementary Figure S4C). We observed that the three estimated time constants are quite similar to those of each individual mHv1 isoform expressed by the *Hvcn1* gene in the mHv1 MDSC current.

All these data are consistent with the three mHv1 isoforms exhibiting distinct voltage-gating activation properties, with the most efficient activation observed in mHv1.1, followed by mHv1.3, and finally, the least efficient activation, mHv1.2. The mHv1 isoforms are expressed and function in combination to produce mHv1 currents in MDSC.

### 2.4 N-terminal beginning length of mHv1 channels could be responsible for mHv1 isoform activity

**FIGURE 4:**
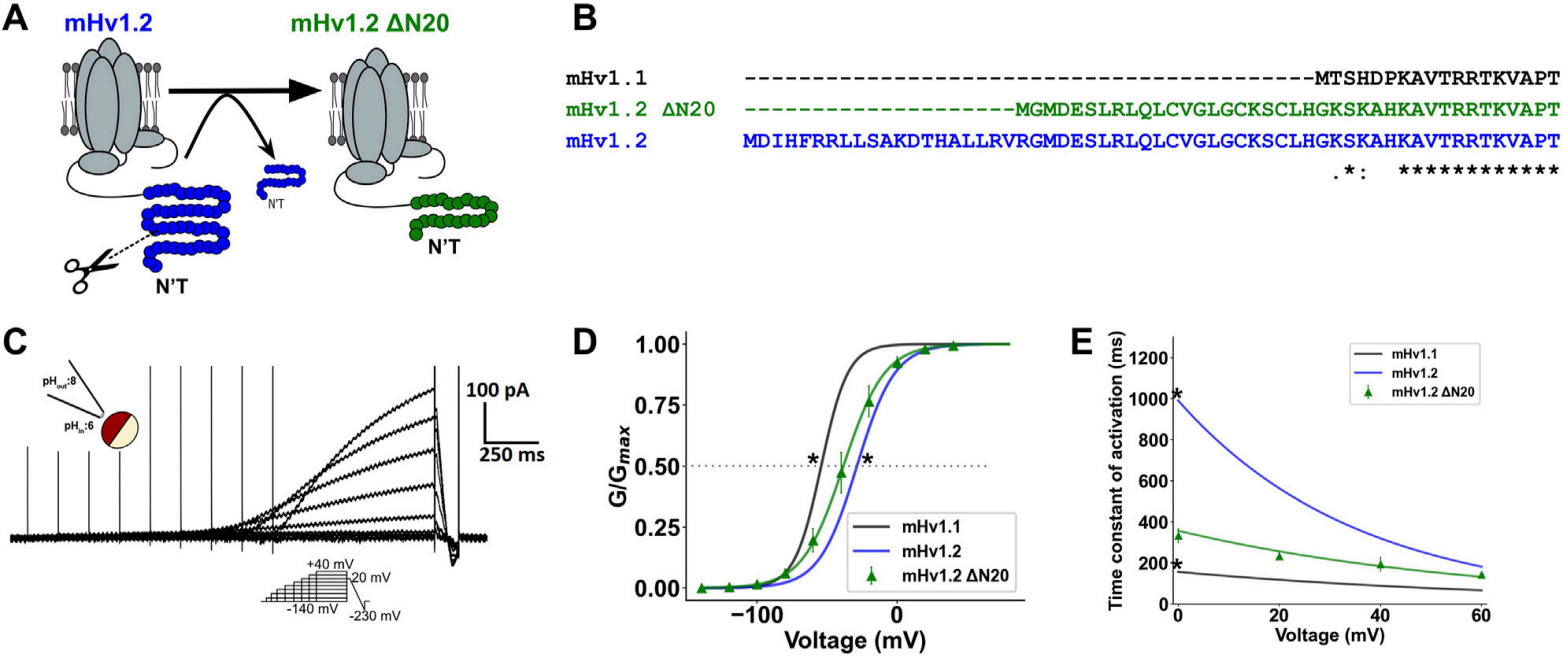
The N-terminal beginning directly modulates gating of mHv1 channels. **(A)** Image representation of the truncation of 20 amino acids from the N-terminal beginning in mHv1.2 to produce mHv1.2 Δ20N. **(B)** Multiple alignment between mHv1.1 and mHv1.2 and its 20-amino acid truncated form, mHv1.2 Δ20N. **(C)** Representative mHv1.2 Δ20N currents, by an inside-out macro-patch clamp at ΔpH = 2. **(D)** Fits shown in Figure 3B at ΔpH = 2 (pHin:6; pHex:8) for mHv1.1 (in black), mHv1.2 (in blue), and normalized conductance for mHv1.2 Δ20N (in green), computed as G (V) = I/(V-Vrev). The data are expressed as mean ± SEM (*n* = 3) and fitted to a Boltzmann function (continuous line), G (V) = Gmax/1+exp [-zδF (V-V_0.5_)/RT] (Student’s t-test; *p < 0.05 for mHv1.1 vs. mHv1.2 Δ20N and mHv1.2 vs. mHv1.2 Δ20N). **(E)** Time constants plotted against voltage for isoforms of mHv1 and mHv1.2 Δ20N obtained from macroscopic Hv1 currents. [Student’s t-test; *p < 0.05 for mHv1.1 vs. mHv1.2 Δ20N and mHv1.2 vs. mHv1.2 Δ20N; results were expressed as mean ± SEM (*n* = 3)].

Thus far, mHv1.1, mHv1.2, and mHv1.3 have displayed distinct activation patterns, which may be influenced by their N-terminal regions. It is noteworthy that all three mHv1 isoforms have varying lengths in their N-terminal segments. Since alternative splicing occurs at the initiation of exon processing, could this imply that the initial segment of the N-terminal region regulates the gating of Hv1 isoforms? We hypothesized that shortening the N-terminal region of mHv1.2 channels would result in a more favorable gating compared to the full-length mHv1.2, while still maintaining less favorable gating compared to mHv1.1. To test this hypothesis, we removed 20 amino acids from the N-terminal region of the mHv1.2 isoform, resulting in a truncated isoform referred to as mHv1.2 Δ20N (a 291-amino acid protein, as depicted in Figures 4A, B).

To confirm the aforementioned findings, we measured voltage-dependent proton currents from inside-out excised patches containing mHv1.2 ΔN20 channels (Figure 4C). The biophysical characterization of this construct was performed, confirming proton selectivity (Supplementary Figure S5A) and showing proton conduction coupled to the movement of six effective charges by a dimeric mHv1 channel (Supplementary Figure S5B).

We found that the GV curve of mHv1.2 ΔN20 was slightly left-shifted in comparison to mHv1.2 GV (*p < 0.05; Figure 4D), supporting the notion that mHv1.2 ΔN20 macroscopic conductance was higher than that of mHv1.2. Additionally, mHv1.2 ΔN20 V_0.5_ was slightly right-shifted compared to mHv1.1 (*p < 0.05; Figure 4D), suggesting that mHv1.2 ΔN20 exhibited less favorable proton conduction compared to mHv1.1.

At the microscopic level, by nonstationary noise analysis of mHv1.2 ΔN20 current fluctuations at ΔpH = 2 and pHi = 6 (Supplementary Figure S5C), we estimated a unitary conductance of 109.3 ± 27.2 fS (*n* = 4), with a maximum open probability of 0.92 ± 0.04 (*n* = 4).

Finally, with regard to mHv1.2 ΔN20 activation kinetics, its τ-V curve (Figure 4E) closely resembled the opening kinetics of mHv1.3, with nonstatistical differences at 0 mV. However, it was faster than mHv1.2 (*p < 0.05) and slower than mHv1.1 (*p < 0.05), suggesting that mHv1.2 ΔN20 activation kinetics was more accelerated than that of mHv1.2 and less accelerated than that of mHv1.1 (Figure 4E).

These findings align with the initial prediction that mHv1.2 ΔN20 gating falls between the mHv1.1 and mHv1.2 phenotypes. Together, these results suggest that the first 20 amino acids of mHv1.2 are involved in the less efficient behavior observed in mHv1.2 gating. These amino acids likely influence either the stabilization of channel closure or the destabilization of the channel opening (Figure 3).

On the other hand, the subsequent 22 amino acids of mHv1.2 ΔN20 seem to contribute to further stabilizing the channel’s closure and/or destabilizing the open state without affecting the activation kinetics (Figure 4).

The canonical isoform, mHv1.1, exhibited the most efficient activation, with the first seven amino acids displaying a positive net charge (Figure 3A). Notably, the beginning of the N-terminal region in mHv1 is closely associated with gating. This is evident in the case of mHv1.3, where the alternative ORF adds four residues and modifies the subsequent seven residues. In total, 11 amino acids with a predominantly negative net charge slow the activation while not affecting the conductance (Figure 3). Therefore, the N-terminal extension and content could be implicated in the differential activation of mHv1 isoforms. Hence, the longer the N terminal end, one should expect a reduced conductance and slowed opening kinetics, as demonstrated by the behavior found in mHv1.2.

## 3 Discussion

Despite the use of mice as a model for studying Hv1 in translational immunology, our understanding of how the *Hvcn1* gene functions and whether isoforms contribute to the diversity of mHv1 remains unclear. This lack of knowledge poses a challenge to investigating mHv1 channels as a potential therapeutic target. It limits our ability to explore genetic and pharmacological approaches that could specifically target Hv1, such as in the case of MDSCs. MDSCs possess the ability to suppress the activation and proliferation of T lymphocytes, and this immunosuppressive function is supported by mHv1 expression and function (Alvear-Arias, J.J., et al., 2022; Cozzolino, M., et al., 2023).

In this study, we gathered a series of evidence that supports the expression of native isoforms of Hv1 in MDSCs of mice. These were expressed by alternative splicing, which tends to favor the expression of the shortest isoform over the larger isoforms in MDSCs. Of the expressed transcripts, one can discern some redundancy when observing that four of the six transcripts, 67% of the transcripts expressed by the *Hvcn1* gene, codify for the mHv1.1 isoform (Figures 1A, B), which is the shortest among mHv1 isoforms in mice. These data strongly suggest that alternative splicing of *Hvcn1* favors the expression of the mHv1.1 isoform in MDSCs. As longer isoforms may require a higher ATP demand for their translation, favoring the expression of the shortest mHv1 isoform could be a metabolic advantage.

As for their function, mHv1 isoforms exhibited features of functional mammalian Hv1 channels, such as voltage and ΔpH-dependence (Figure 2A), a unitary conductance in the order of the fS (Figure 2B), proton selectivity (Figure 2D), and dimeric state (Supplementary Figure S3). Interestingly, the longer isoforms exhibit a less efficient gating compared to mHv1.1, corresponding to the length of their N-terminal regions; for example, mHv1.3, the mid-length isoform, exhibited slowed activation kinetics (Figure 3C), and mHv1.2, the longest isoform, exhibited both slowed activation kinetics and a GV curve shifted toward depolarized potentials (Figure 3C).

It appears that the N-terminal domain of Hv1 channels acts as a regulating site for a diversity of genetic and/or post-translational processes, such as naturally occurring mutations (M91T in hHv1, Lovannisci, Illek, B., & Fischer, H., 2010), phosphorylation (Thr29 in hHv1, Musset, B., et al., 2010), physiological proteolysis (Arg68 in hHv1, Berger, T. K., Fußhöller, D. M., et al., 2017), or the alternative splice production of HVCN1_S_ (lacking 20 amino acids at the beginning of the N terminal compared to full-length HVCN1_L_). mHv1.1 is the ortholog of HVCN1_L_ in mice, where the longer mHv1 isoforms exhibit the addition of amino acids at the beginning of the N-terminal domain, contrary to HVCN1_S_, which lacks the first 20 amino acids, while exhibiting activation kinetics slightly slower than that of HVCN1_L_ (Hondares et al., 2014). In the case of the N-terminal region of mHv1 isoforms, the addition of specific amino acids is associated with a less efficient gating of Hv1. For instance, mHv1.3 exhibits the addition of four amino acids and a modification of the subsequent seven amino acids compared to mHv1.1. This alteration results in slower activation kinetics (Figure 3C). Additionally, the addition of 42 amino acids and modifications in six out of the next seven amino acids further hinders the gating of mHv1. Compared to mHv1.1 or even mHv1.3, this results in a more depolarized V0.5 and slower activation kinetics (Figure 3).

**FIGURE 5:**
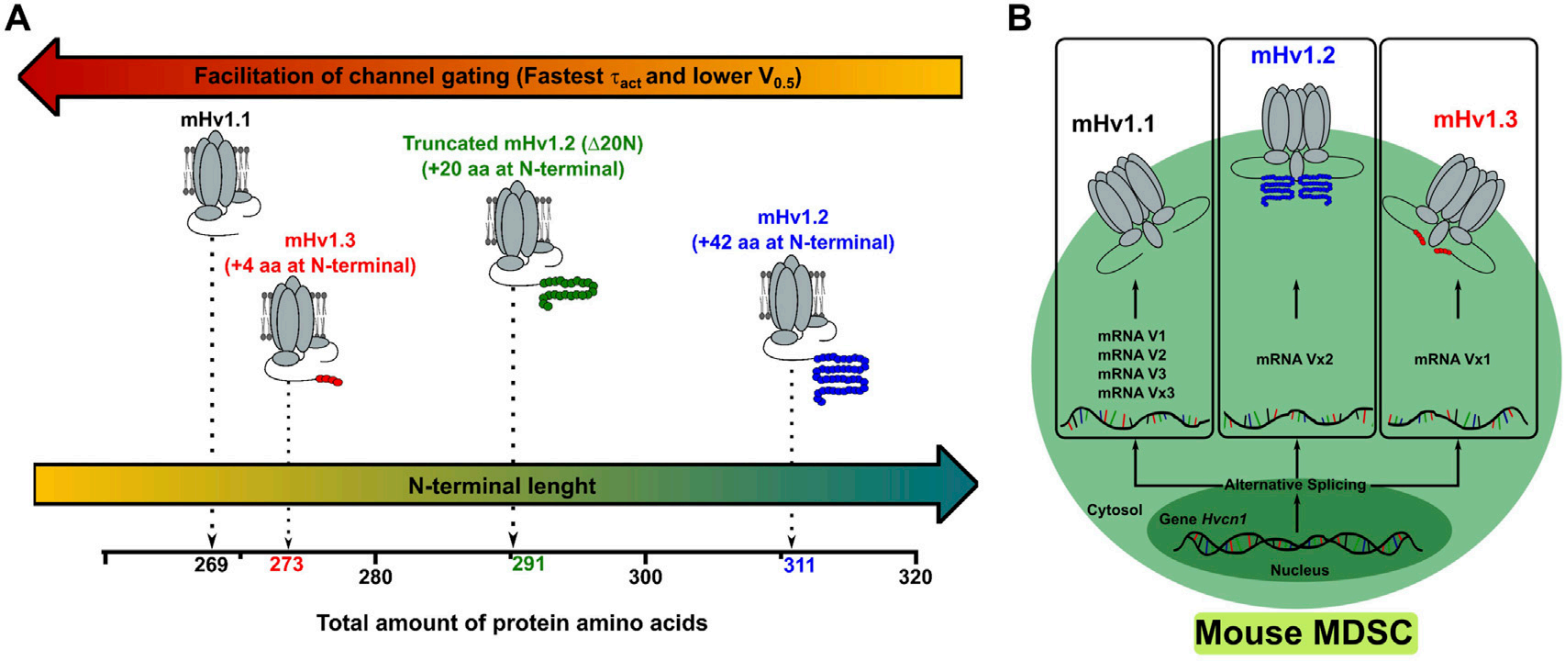
The Hvcn1 gene of MDSCs expresses up to three functional isoforms alternatively spliced at their N terminal. **(A)** The data obtained in the characterization of the macroscopic currents of the different isoforms of mHv1 allow us to conclude that a shorter N terminal promotes the efficiency of the channel gating. Thus, depending on the functional requirements, the MDSCs could modulate the activity related to proton extrusion by preferring isoforms with N terminals of different lengths. **(B)** Image representation of an MDSC expressing the Hvcn1 gene and alternatively splicing different transcripts that express as mHv1 isoforms. The alternative splicing regulates the extension of the N-terminal portion of mHv1 isoforms, which regulates the conductance of protons and opening kinetics in these channels.

In light of the previous observations, we investigated whether changing the extension of the N terminal could regulate the Hv1 channel’s function. We hypothesized that by shortening the N terminal of the longest mHv1 isoform by 20 amino acids, the truncated short Hv1 construct (mHv1.2 ΔN20) would adopt a gating phenotype between mHv1.2 and mHv1.3 (Figures 4A–C). We found that mHv1.2 ΔN20 exhibited a shift of the GV to the left compared to mHv1.2 GV, while still exhibiting a GV shifted to the right compared to mHv1.3 GV (Figure 4D) with its τ-V curve falling right next to mHv1.3 τ-V (Figure 4E), confirming the prediction. With this, we demonstrated that in mHv1 isoforms, shortening the N terminal beginning leads to a more efficient activation. Thus, extending the N terminal causes a less efficient activation (Figure 5A).

When evaluating the secondary structure of the mHv1 isoforms, we observed that the additional 42 residue additions in mHv1.2 led to the folding of four helices, separated by random coils (Supplementary Figure S6A, middle), and four extra residues in mHv1.3 brought about the folding of a helix (Supplementary Figure S6A, bottom), which lacks in mHv1.1 (Supplementary Figure S6A, top). In mHv1.1, the first 20 amino acids are in high proportion, forming a random coil (Supplementary Figure S6A, top), which may contribute to accelerating the opening kinetics, as the deletion observed in HVCN1_S_ leads to a slowing of their activation kinetics when compared to HVCN1L or mHv1.1. *Ab initio* models of the N-terminal domains of mHv1 isoforms show consistency with the predicted secondary structure (Supplementary Figure S6B). mHv1.2 ΔN20 lacks the two initial globular helixes present in mHv1.2 (Supplementary Figure S6A, middle). These two helixes could be implicated in slowed activation kinetics when compared to mHv1.1. This suggests that the length of the N terminal contributes to the gating efficiency of Hv1 channels (Figure 4; Figure 5A), where, in general, a longer N terminal will shift the GV curve toward more depolarizing voltages, destabilizing the open state and slowing its opening.

We conclude that, in MDSCs, the alternative splicing of the *Hvcn1* gene leads to the modulation of the mHv1 function by directly modulating the extension of the N-terminal beginning (Figure 5), which is tightly coupled to the gating of mHv1 channels, where the shortest isoform exhibits the most advantageous gating and the longest isoform exhibits the least advantageous gating (Figure 5). Given that mHv1.1 expression appears to be dominant and that mHv1.1 exhibits the most efficient gating phenotype among mHv1 diversity, it would be interesting to explore in more detail molecules that are able to interact and induce changes in the function of mHv1.1, such as Hv1 classic activators (i.e., arachidonic acid) (Kawanabe and Okamura 2016), but more significantly, inhibitors (i.e., zinc ion and guanidine ion) (Alvear-Arias et al., 2022, Alvear-Arias et al., 2023), and genetic approaches directed to diminish the levels of mRNAs that translate as mHv1.1, such as siRNA or CRISPR-Cas. mHv1.1 could potentially not only be used as a biomarker, but more promisingly, also as a therapeutic target in MDSC, with the aim to alleviate immunosuppression in cancer contexts.

## 4 Materials and methods

### 4.1 Bioinformatics

By the ClustalW^®^ program (https://www.genome.jp/tools-bin/clustalw, Thompson et al., 1994), multiple alignments for sequences reported at the NCBI database (https://www.ncbi.nlm.nih.gov/gene/74096) corresponding to the Hv1 transcripts or their open-reading frame were performed to determine similitudes among sequences and define structural motifs. A putative alternative edition among transcripts was predicted using the Splign program (https://www.ncbi.nlm.nih.gov/sutils/splign/splign.cgi). Query blast and identity percentage were calculated using BLAST-p (https://blast.ncbi.nlm.nih.gov/Blast.cgi). Primers used to amplify the full transcript or the codifying of the sequence (Supplementary Table S1) were designed using AmplifX 2^®^ (version 2.0.6) software, and their selectivity was evaluated by the Primer-BLAST program (https://www.ncbi.nlm.nih.gov/tools/primer-blast/) using the RefSeq mRNA database *for M. musculus* as the target organism. Prediction of secondary structure was done by using PSIPRED v4.0 (http://bioinf.cs.ucl.ac.uk/psipred, Jones DT, 1999). The mHv1 isoform N terminal structure was obtained by performing de novo structure prediction with the Rosetta AbinitioRelax application (Bradley et al., 2005). The models with the lowest pseudo-energy score, selected from a range of 5,000 to 10,000 models, were chosen as representatives for N-terminal beginning isoforms and drawn using VMD (Humphrey et al., 1996), with licorice representing the beginning of the protein (methionine, white) and the last residue (Arg, Lys, and Arg for mHv1.1, mHv1.2, and mHv1.3, respectively, blue).

### 4.2 Animals, cell culture, and MDSC differentiation

Mice C57BL/six mice (8–12 weeks old) were maintained in the University of Valparaiso bioterium (Valparaiso, Chile). All experiments followed the institutional guidelines of the University of Valparaiso’s Animal Care and Use Committee. Bone marrow-derived cells (BM cells) were isolated from the femurs and tibias of C57BL/6 mice and cultured to obtain MDSCs following the protocol previously described by Alvear-Arias et al. (2022). Cellular differentiation was obtained 4 days after incubating 2.5 × 10^5^ BM cells with 40 ng/mL of GM-CSF at 37°C in a humidified atmosphere of 5% CO_2_.

### 4.3 Total RNA extraction and RT-PCR

Once MDSC differentiation was observed, cells were detached from the Petri dish using 2 mM PBS/EDTA and then centrifuged at 200 g for 5 min to re-suspend them in PBS on ice. Total RNA was extracted using the RNA-Solv kit (OMEGA Biotek) and lysed using a 20G needle and syringe. To degrade genomic DNA, samples were treated with dsDNAseI (ThermoFisher Scientific). cDNA was synthesized using the Maxima H Minus First-Strand cDNA Synthesis Kit (ThermoFisher Scientific) with 0.7–1 µg of total RNA. PCR and overhang PCR (oPCR) reactions were performed according to the instructions of the Platinum SuperFi DNA Polymerase Kit (ThermoFisher Scientific) in a final volume of 20 µL. The PCR products were run on a 1% agarose gel, stained with GelStar (Lonza) 2X, and visualized using a 3UV transilluminator (Epi Chemi Darkroom). The O’Gene Ruler 1 Kb DNA Ladder (ThermoFisher Scientific) was used as a molecular weight marker.

### 4.4 Cloning and heterologous expression

First, the oPCR products were purified using the PCR purification kit (Qiagen), and then they were site-directly cloned into the BamHI/HindIII restriction sites from the pGEM-3 vector. After identifying Hv1 transcripts by Sanger nucleotide sequencing, selected clones were linearized with the BamHI restriction enzyme to perform *in vitro* transcription and obtain cRNA, following the instructions of the T7 Message Machine Kit (Thermo Scientific). Finally, the cRNA was quantified and visualized by electrophoresis using agarose. cRNA was stored at −80°C until use. For mHv1 isoforms, 50 nL of cRNA at concentrations ranging from 0.5–4 μg/μL were injected into *X. laevis* oocytes. The oocytes were incubated at 12–25°C in ND96 or SOS medium, supplemented with antibiotics and 2–5 mM pyruvate, for approximately 24–80 h post-injections before recording.

### 4.5 Electrophysiology

Borosilicate glass (World Precision Instruments, Inc.) used as recording micropipettes was pulled using a P-97 Flaming/Brown automatic pipette puller (Sutter Instruments, Co.) and polished in an MF-830 Microforge (Narishige, Co.). Micropipettes used for inside-out patch clamp recordings in oocytes had diameters ranging from 3 to 15 μm, with resistances between 1 and 8 MΩ, while micropipettes used for whole-cell patch clamp recording in MDSCs had a resistance of 7–12 MΩ. The Ag-AgCl reference electrode was connected to the bath solution through an agar bridge with 3M KCl solution and ground. The registered currents were obtained using an Axopatch 200B amplifier (Axon Instruments) by applying voltage protocols (pCLAMP, version 10.3) acquired at 250 kHz, low-pass-filtered at 10 kHz (Frequency Devices), and analog-to-digital-converted using a Digidata 1550 (Axon instruments). Solutions used for proton current recordings in MDSCs for both bath and pipette were as follows: 100 mM MES buffer, 30 mM tetraethylammonium hydroxide (TEAOH), 2 mM magnesium chloride (MgCl_2_), 1 mM ethylene glycol-bis (b-aminoethyl) N, N, N′, N″- tetra acetic acid (EGTA), and 160 mM N-methyl-D-glucamine (NMDG)—methane sulfonic acid (MeSO_3_) to reach an osmolarity of ∼300 mOsm for solutions at pH 5.5 and 100 mM HEPES buffer, 30 mM TEAOH, 2 mM MgCl_2_, 2 mM MgCl2, 1 mM EGTA, and 160 mM NMDG-MeSO_3_ (or solutions at pH 7.5). The solution used for pharmacological perfusion during proton current recordings contained 10 mM ZnCl_2_, 100 mM HEPES buffer, 30 mM TEAOH, 2 mM MgCl_2,_ and 160 mM NMDG-MeSO_3_ at pH 7.5. Solutions used for proton current recordings in oocytes injected with cloned transcripts include 110 mM HEPES, 1 mM EGTA, 2 mM MgCl_2_, and 110 mM NMDG + MeSO_3_ at pH 5, 5, 7, and 5, respectively. All electrophysiology experiments were performed at room temperature (∼21°C).

Macroscopic currents of mHv1 isoforms were obtained at the end of each depolarization pulse, which can be expressed as conductance according to Ohm’s law:

$$I_{H^{+}}=G_{H^{+}}\left(V-V_{H^{+}}\right),$$ (1)

where $G_{H^{+}}$ represents the macroscopic conductance of protons and $\left(V-V_{H^{+}}\right)$ represents the electrical driving force, with V the voltage and $V_{H^{+}}$ the reversal potential of protons. To build current–voltage (IV) plots, the current values were plotted against the potential (I–V curve) and fitted to a normalized

Boltzmann function:

$$I_{H^{+}=}\frac{I_{\max}}{1+\exp\frac{z\delta F\left(V-V_{0.5}\right)}{RT}}.$$ (2)

Here, I_max_ represents the maximum current, zδ represents the voltage dependence, F is the Faraday constant, V is the voltage, V_0.5_ is the voltage at which 50% of the channels are open, R is the universal gas constant, and T is the temperature in Kelvin.

For nonstationary noise analysis, current ensembles (ranging from 11 to 256) from isoforms of Hv1, expressed in membranes of *X. laevis* oocytes, were used to calculate the mean current and variance. These calculations followed procedures described elsewhere (Alvarez et al., 2002) and were fitted using the following parabolic equation:

$$\sigma^{2}\left(t\right)=i<I\left(t\right)>-\frac{{<I\left(t\right)>}^{2}}{N},$$ (3)

where $\sigma^{2}\left(t\right)$ represents the variance, i is the unitary current, $<I\left(t\right)>$ is the mean current, and N is the number of channels in the membrane patch.

For the measurement of reversal potential, it is generally defined as follows:

$$V_{rev}=\frac{\sum E_{i}g_{i}}{\sum g_{i}}.$$ (4)

In the previous equation, E_i_ represents the reversal potential of the ion i, and g_i_ is the conductance of the ion i. Using solutions with no sodium and potassium chloride, Eq. 4 transforms into the reduced form of the reversal potential equation as follows:

$$V_{rev}=V_{H^{+}},$$ (5)

where $V_{H^{+}}$ can be described by the following Nernst potential for protons:

$$V_{H^{+}}=\frac{RT}{zF}\ln\frac{{\left[H\right]}_{ex}^{+}}{{\left[H\right]}_{in}^{+}},$$ (6)

leading to the conclusion that the membrane is exclusively selective for the permeation of protons.

The experimental determination of the reversal potential can be achieved by observing the intersection of fast ramp currents. The intersection of these currents can be represented by the expanded form of the total current as follows:

$$I=I_{H^{+}}+I_{Leak}+I_{Capacitive}=G_{H^{+}}\left(V-V_{H^{+}}\right)+G_{Leak}\left(V-V_{Leak}\right)+C\frac{dV}{dt},$$ (7)

where the macroscopic total current (I) is equal to the sum of the proton current $I_{H^{+}}$ (described in Eq. 1), the leak current $I_{Leak}$ , and the capacitive current, $I_{Capacitive}$ , with $G_{Leak}$ as the leak conductance, $V_{Leak}$ as the equilibrium potential of the leak, and C as the capacitance of the membrane. It is of utmost importance to note that capacitive currents were considerably lowered on-line by using the amplifier circuitry, leading $I_{Capacitive}$ to a diminished, but constant, remnant capacitive current, and that leak currents correspond to an artifact that may come from an imperfect seal that favors the leak of ions. As a consequence, G_leak_ can be very large or very small depending on the patch nature, but such values were corrected off-line by leak subtraction, leading $I_{Leak}$ to transform into a constant remnant leak current, $I_{const}$ . These corrections transform Eq. 7 into

$$I\approx I_{H^{+}}+I_{const}.$$ (8)

When the voltage ramp matches $V_{H^{+}}$ , $\left(V-V_{H^{+}}\right)$ is 0, as does $I_{H^{+}}$ , leading Eq. 8 to become

$$I\approx I_{const}.$$ (9)

Therefore, at ${V=V}_{H^{+}}$ , all the currents induced by the voltage ramp will cross each other at the same point.

$$I_{const}\left(V_{i}\right)\approx I_{const}\left(V_{i+1}\right).$$ (10)

Macroscopic measurements of mHv1 isoform currents were performed by using variable-duration pulse protocols, applied to diminish the proton depletion (Carmona et al., 2021; Zhao and Tombola, 2021; Alvear-Arias et al., 2022). All current traces were followed by a fast ramp. The variance of the currents elicited by the fast ramp was obtained to determine the time at which the currents were reversed. The minimum value of variance indicates V = $V_{H^{+}}$ .

For the limiting slope method, slow and symmetrical voltage ramps (0.5 mV/s–1 mV/s) were used. Currents were leak-subtracted off-line and filtered to 10 Hz when external noise was contaminating the currents. The data points were decimated (average of five points) and mean-binned (100 bins).

To estimate the effective charge coupled to the channel opening, a log-linear plot of the conductance vs. voltage was built, and the data were fitted to the equation at low open conductance values (at hyperpolarized potentials) as follows:

$$G\left(V\right)=G0\exp\left(-\frac{z\delta FV}{RT}\right).$$ (11)

In Eq. 7, G_0_ represents a pre-exponential conductance factor. Subsequently, zδ was plotted against voltage using the following relationship:

$$z\delta \left(V\right)=\frac{kT}{e_{0}}\;\frac{d}{dV}lnG\left(V\right).$$ (12)

In Eq. 8, k is the Boltzmann’s constant and e_0_ is the elementary charge.

For calculation of proton conductances, tail currents were transformed into conductance values measured from isochrones taken at the tail of the currents, where

$$I_{H^{+}}=G_{H^{+}}.$$ (13)

All the conductance values were plotted against the potential and fitted to a normalized Boltzmann function.

$$G_{H^{+}=}\frac{G_{\max}}{1+\exp\frac{z\delta F\left(V-V_{0.5}\right)}{RT}}.$$ (14)

G_max_ represents the maximum conductance. In a two-state channel model, the time constants for activation α (V) and deactivation β (V) can be represented as follows:

$$\tau \left(V\right)=\frac{1}{\alpha \left(V\right)+\beta \left(V\right)},$$ (15)

where α (V) can be isolated from α (V) +β (V) at depolarized potentials, transforming into

$$\tau_{act}\left(V\right)=\frac{1}{\alpha \left(V\right)}.$$ (16)

mHv1 isoform proton currents were generated by shortened-depolarizing voltage protocols coupled with a fast ramp and evaluated to determine if there was depletion of protons by checking the reversal potential. The time constant for mid-activation, τ_act_, was obtained by fitting the previous macroscopic ON currents to a standard exponential function:

$$I\left(t\right)=\alpha \left(0\right)\exp\frac{-\tau_{act}}{t}+C.$$ (17)

In the aforementioned equation, α (0) is the time constant at 0 mV, t represents the time, and C is a constant value. Assuming that MDSCs express the three mHv1 isoforms and that, at 0 mV, all these isoforms exhibit the same activation kinetic differences, then MDSC Hv1 currents at 0 mV were reproduced by the sum of three exponential functions:

$$I\left(t\right)=\alpha_{1}\left(0\right)\exp\frac{-\tau_{act,1}}{t}+\alpha_{2}\left(0\right)\exp\frac{-\tau_{act,2}}{t}+\alpha_{3}\left(0\right)\exp\frac{-\tau_{act,3}}{t}+C.$$ (18)

Each exponential returned three time constants, representing the presence of each mHv1 isoform. All the data were analyzed using Clampfit 10.7, GraphPad Prism 6.0, and custom-made Python 3.8 scripts. Statistics are presented as mean ± standard error of the mean (SEM), unless otherwise reported.

## Data Availability

The datasets presented in this study can be found in online repositories. The names of the repository/repositories and accession number(s) can be found in the article/Supplementary Material.
