# Supplementary material for: N-terminal region is responsible for mHv1 channel activity in MDSCs
Source: Front Pharmacol. 2023 Oct 17;14:1265130. doi: 10.3389/fphar.2023.1265130 (PMC10616795; doi:10.3389/fphar.2023.1265130)
Supplement: Supplementary file 1 [file DataSheet1.pdf]

A

```
Variant 2   CAGTTCTCTCACTGTTGGATCTTGAGACAACAGGAAAATGACTTCCCATGACCCAAAGGC
Exon6       CAGTTCTCTCACTGTTGGATCTTGAGACAACAGGAAAATGACTTCCCATGACCCAAAG--
            **********************************************************

Exon9       ----------------------------------------------------------GC
                                                                      **
```

B

```
Variant x1  AACTGGCCCAGACAGCAGCAGTAACACAGACGACACTGTTTATCAACCCGTATCCATGAA
Exon8       AACTGGCCCAGACAGCAGCAGTAACACAGACGACACTGTTTATCAACCCGTATCCATGAA
            ************************************************************

Exon9       ------------------------------------------------------------

Variant x1  TGGACATTCATTTTCGAAGGTTGCTTTCAGCAAAGGACACACATGCGTTGTTGCGGGTCA
Exon8       TGGACATTCATTTTCGAAGGTTGCTTTCAGCAAAGGACACACATGCGTTGTTGCGGGTCA
            ************************************************************

Exon9       ------------------------------------------------------------

Variant x1  GGGGCATGGATGAGTCCTTAAGATTACAGCTGTGTGTTGGCTTAGGTTGTAAAAGCTGTT
Exon8       GGGGCATGGATGAGTCCTTAAGATTACAGCTGTGTGTTGGCTTAGGTTGTAAAAGCTGTT
            ************************************************************

Exon9       ------------------------------------------------------------

Variant x1  TGCACGGGAAATCCAAAGCACATAAGGCCGTCACTCGCAGAACCAAGGTGGCTCCCACCA
Exon8       TGCACGGGAAATCCAAAGCACATAAG----------------------------------
            **************************

Exon9       --------------------------GCCGTCACTCGCAGAACCAAGGTGGCTCCCACCA
                                      **********************************
```

C

```
Variant x2  AGCAAGCTCCTGGGGCAAAGGTCTTGAGGCCTGGAACACAGGCTGAATGGAAACAGAATG
Exon7       AGCAAGCTCCTGGGGCAAAGGTCTTGAGGCCTGGAACACAGGCTGAATGGAAACAGAATG
            ************************************************************

Exon9       ------------------------------------------------------------

Variant x2  GCCACGCAGGGAGAGTCAGGCCGTCACTCGCAGAACCAAGGTGGCTCCCACCAAGAGGAT
Exon7       GCCACGCAGGGAGAGTCAG-----------------------------------------
            *******************

Exon9       -------------------GCCGTCACTCGCAGAACCAAGGTGGCTCCCACCAAGAGGAT
                               *****************************************
```

D

```
mHv1.1   ----------------------------------------MTSHDPKAVTRRTKVAPT
mHv1.2   MDIHFRRLLSAKDTHALLRVRGMDESLRLQLCVGLGCKSCLHGKSKAHKAVTRRTKVAPT
mHv1.3   --------------------------------------METEWPRRESQAVTRRTKVAPT
                                                   :   :***********

mHv1.1   KRMSRFLKHFTVVGDDYHTWNVNYKKWENEEEEEEPAPTSAEGEGNAEGPDAEAGSASTP
mHv1.2   KRMSRFLKHFTVVGDDYHTWNVNYKKWENEEEEEEPAPTSAEGEGNAEGPDAEAGSASTP
mHv1.3   KRMSRFLKHFTVVGDDYHTWNVNYKKWENEEEEEEPAPTSAEGEGNAEGPDAEAGSASTP
         ************************************************************
                           Cytoplasmic Domain (CD)

mHv1.1   RQSLDFRSRLRKLFSSHRFQVIIICLVVLDALLVLAELLLDLKIIEPDEQDYAVTAFHYM
mHv1.2   RQSLDFRSRLRKLFSSHRFQVIIICLVVLDALLVLAELLLDLKIIEPDEQDYAVTAFHYM
mHv1.3   RQSLDFRSRLRKLFSSHRFQVIIICLVVLDALLVLAELLLDLKIIEPDEQDYAVTAFHYM
         ************************************************************
                           Transmembrane Segment (TS) 1

mHv1.1   SFAILVFFMLEIFFKIFVFRLEFFHHKFEILDAFVVVVSFVLDLVLLFKSHHFEALGLLI
mHv1.2   SFAILVFFMLEIFFKIFVFRLEFFHHKFEILDAFVVVVSFVLDLVLLFKSHHFEALGLLI
mHv1.3   SFAILVFFMLEIFFKIFVFRLEFFHHKFEILDAFVVVVSFVLDLVLLFKSHHFEALGLLI
         ************************************************************
              TS 2                         TS 3

mHv1.1   LLRLWRVARIINGIIISVKTRSERQILRLKQINIQLATKIQHLEFSCSEKEQEIERLNKL
mHv1.2   LLRLWRVARIINGIIISVKTRSERQILRLKQINIQLATKIQHLEFSCSEKEQEIERLNKL
mHv1.3   LLRLWRVARIINGIIISVKTRSERQILRLKQINIQLATKIQHLEFSCSEKEQEIERLNKL
         ************************************************************
              TS 4                                Coiled-Coil Region (CCR)

mHv1.1   LKQNGLLGDVN
mHv1.2   LKQNGLLGDVN
mHv1.3   LKQNGLLGDVN
         ***********
```

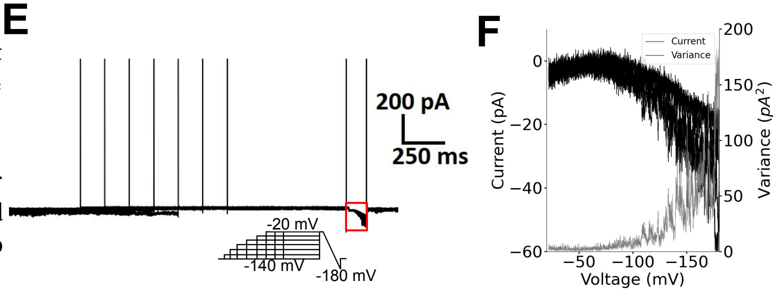

Supplementary Figure 1. The Hvcn1 gene is predicted to express mRNA transcript with different open reading frames. **(A), (B) and (C)** Multiple alignment between the exonic composition of the transcript and the variant for V_2, Vx1 and Vx2, respectively. The blue mark in each transcript points to the beginning of the open reading frame (ORF), starting for the initial ATG. **(D)** Multiple alignment of the three predicted isoforms. All these proteins exhibit a very high conservation among their sequences following the initial changes in their N terminal domain. **(E)** Non-injected oocyte registered at ΔpH=2 (pHin=6, pHout=8), which shows no detectable proton currents. **(F)** Current from fast ramp highlighted in the red box in **(E)**. No reversion of the currents is detected.

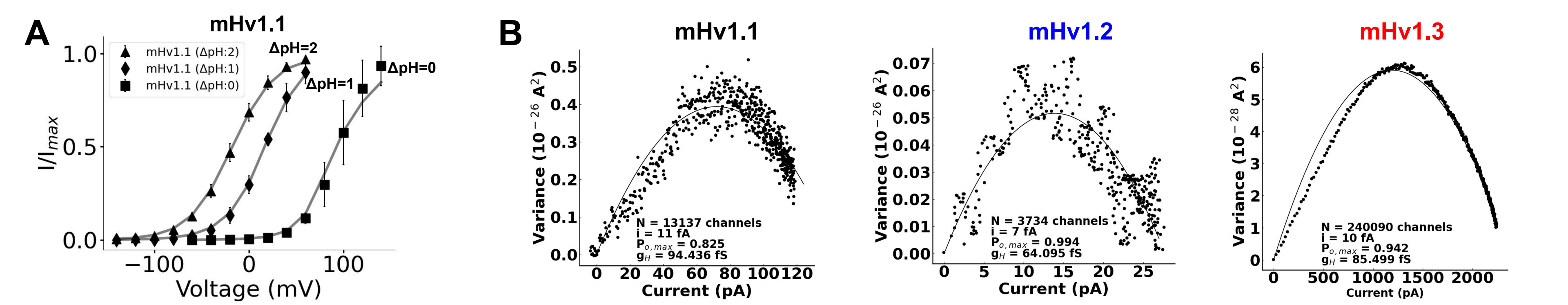

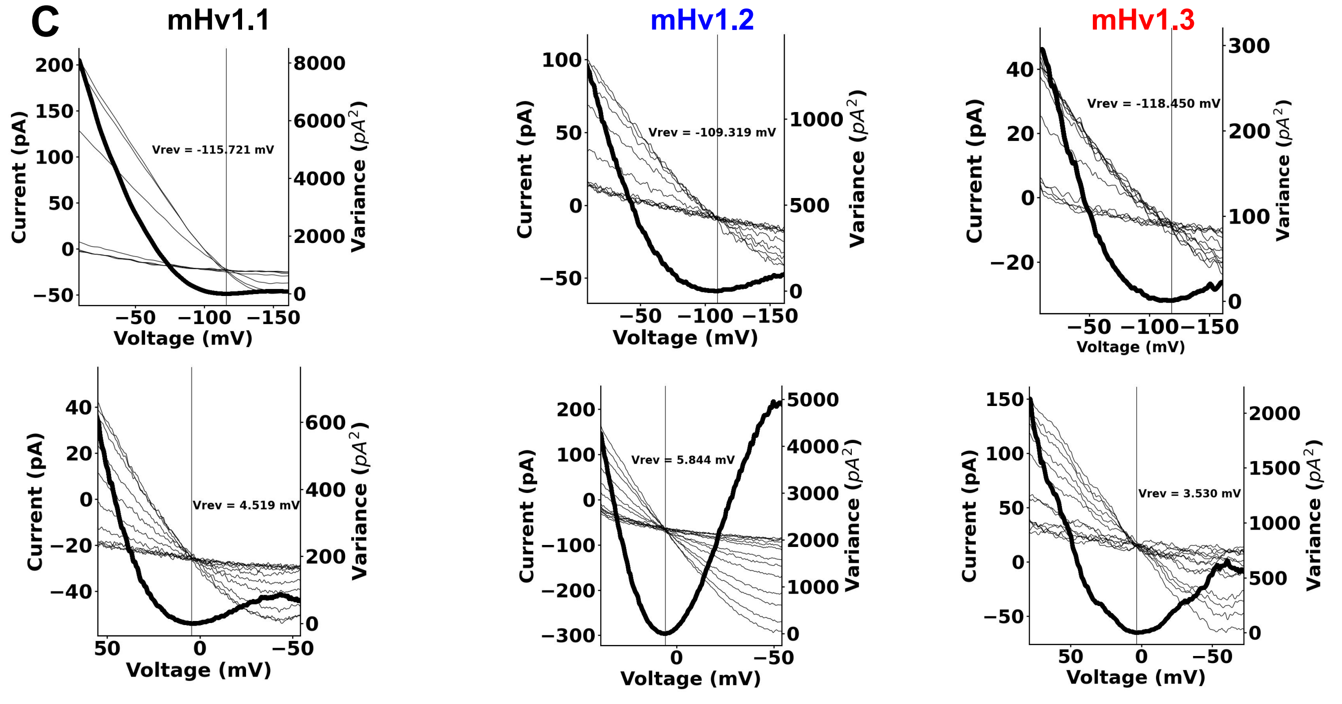

Supplementary Figure 2. Continuation of biophysical characterization of mHv1 isoforms. (A) ΔpH-dependence of the IV curve for mHv1.1 (data in squares are at ΔpH=2 (pHin=6, pHout=8), diamonds at ΔpH=1 (pHin=6, pHout=7), and triangles at ΔpH=0 (pHin=7, pHout=7)). **(B)** Non-stationary noise analysis for mHv1 isoforms at ΔpH=2 (pHin=6, pHout=8), and at 0 mV. The number of channels (N), unitary current (i), maximum open probability (PoMax), and unitary conductance (gH) are reported at the insets in each graph. **(C)** Reversal potential estimation for mHv1.1, mHv1.2 and mHv1.3 at ΔpH:2 (top), and ΔpH:0 (bottom). Variance is shown as bold curves.

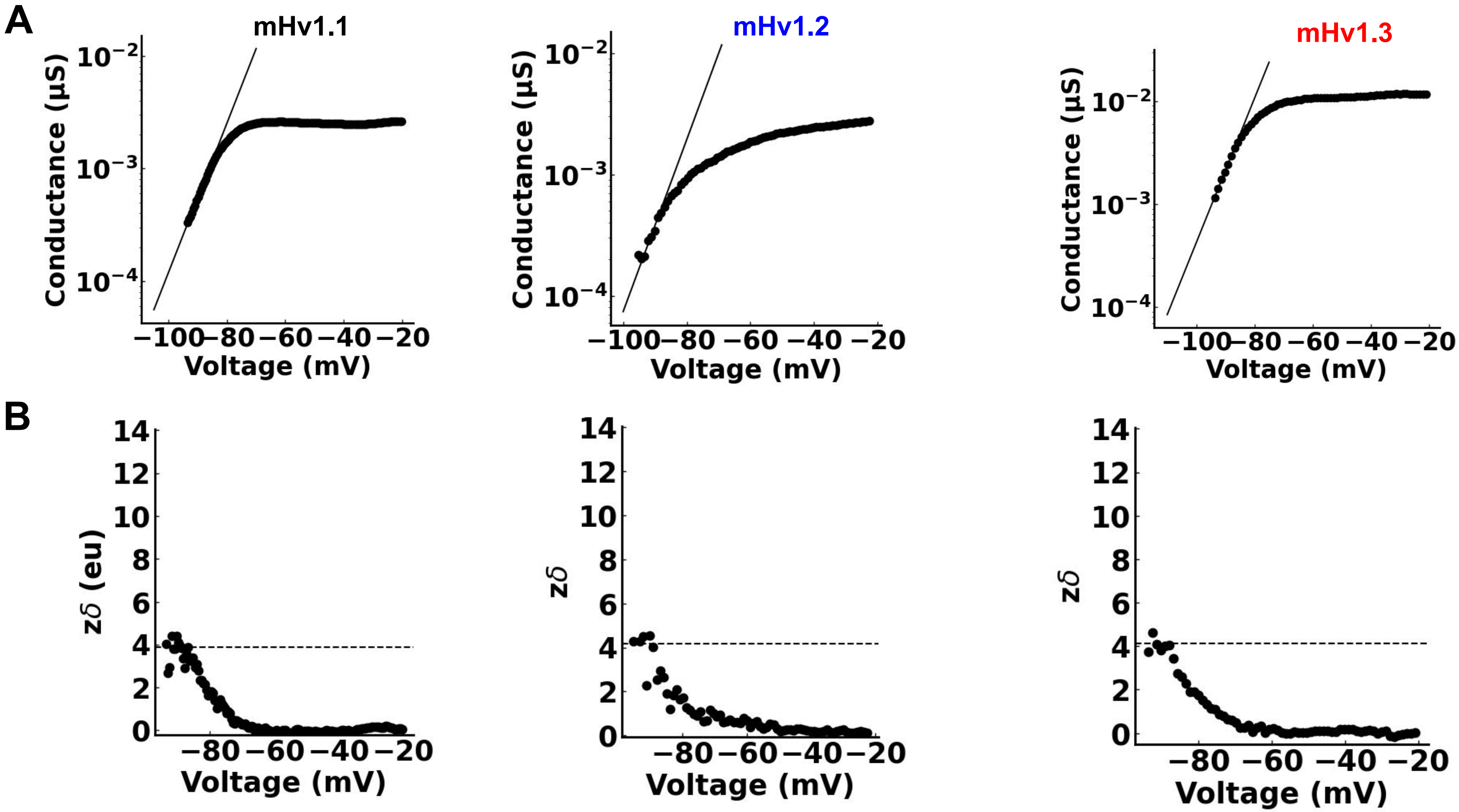

Supplementary Figure 3. The novel cloned longer isoforms of Hv1 exhibits gating cooperativity. (A) mHv1 isoforms currents at ΔpH=2 (pHin=6, pHout=8) were transformed into conductances and fitted at hyperpolarized potentials with the Boltzmann function: G = G0*exp(-zδFV/RT)**. (B)** The charge coupled to the opening for all mHv1.1 isoforms, measured at different potentials as they eventually reach a stationary value of charge, exactly before its curve changes abruptly in the limiting slope. The effective gating charge coupled to the opening of mHv1.1, mHv1.2 and mHv1.3 was: 4.19 ± 0.13, 4.30 ± 0.18, and 4.44 ± 0.17 , respectively (n=3 for each isoform; data as mean ± S.E.M.).

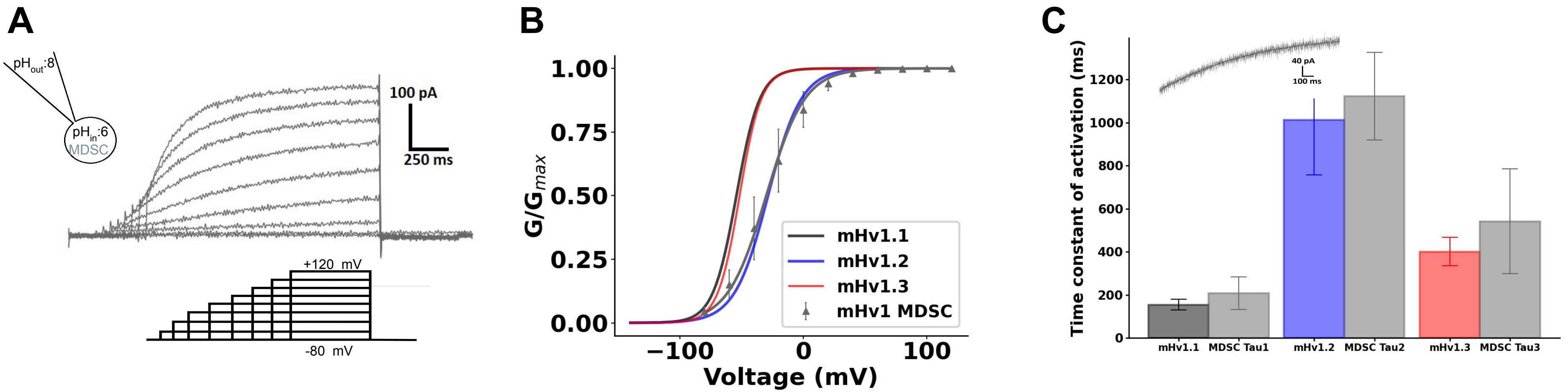

Supplementary Figure 4. MDSC expresses functional isoforms of mHv1 (A) Representative Hv1 family currents from MDSC in response to a depolarization voltage protocol (inset:left) at ΔpH:2 (pHin=6; pHex=8). Scale is shown at inset:right. **(B)** Normalized GVs from mHv1 in MDSC at ΔpH=2 (dimgrey triangles), fitted with the Boltzmann equation: $G(V)=Gmax/1+exp(-z\delta F(V-V_{0.5})/RT)$, plotted with the fits obtained in Figure 3 for mHv1 isoforms in oocytes from *X. laevis*. **(C)** Time constant of activation at 0 mV and ΔpH=2, estimated by a summatory of three exponential functions for MDSC currents (expressed as MDSC Tau1, MDSC Tau2 and MDSC Tau3; expressed as average ± SEM, n=3, fit at the inset; see Methods) compared to all three mHv1 isoforms.

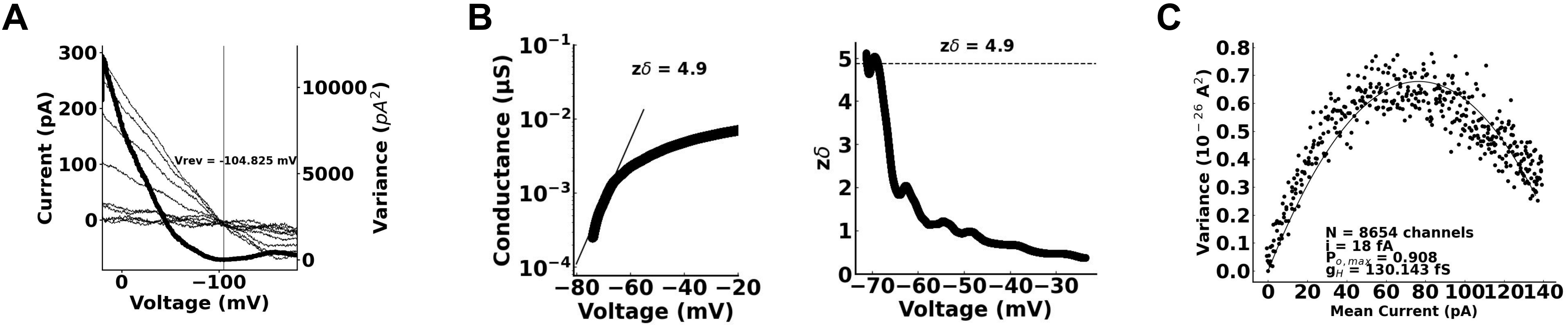

Supplementary Figure 5. Δ20Naa mHv1.2 exhibited native properties of functional mHv1 channels. (A) Reversal potential estimation showing selectivity to protons at ΔpH=2 (pHin=6, pHout=8) of Δ20Naa mHv1.2 channels. **(B)** Estimation of the charge coupled to the opening of Δ20Naa mHv1.2 channels at ΔpH=2 (pHin=6, pHout=8), showing a value consistent with a dimer oligomeric state. **(C)** Non-stationary noise analysis on an ensemble of 100 consecutive depolarizing pulses at 20 mV at ΔpH=2 (pHin=6, pHout=8) The number of channels (N), unitary current (i), maximum open probability (PoMax), and unitary conductance (gH) are reported at the inset.

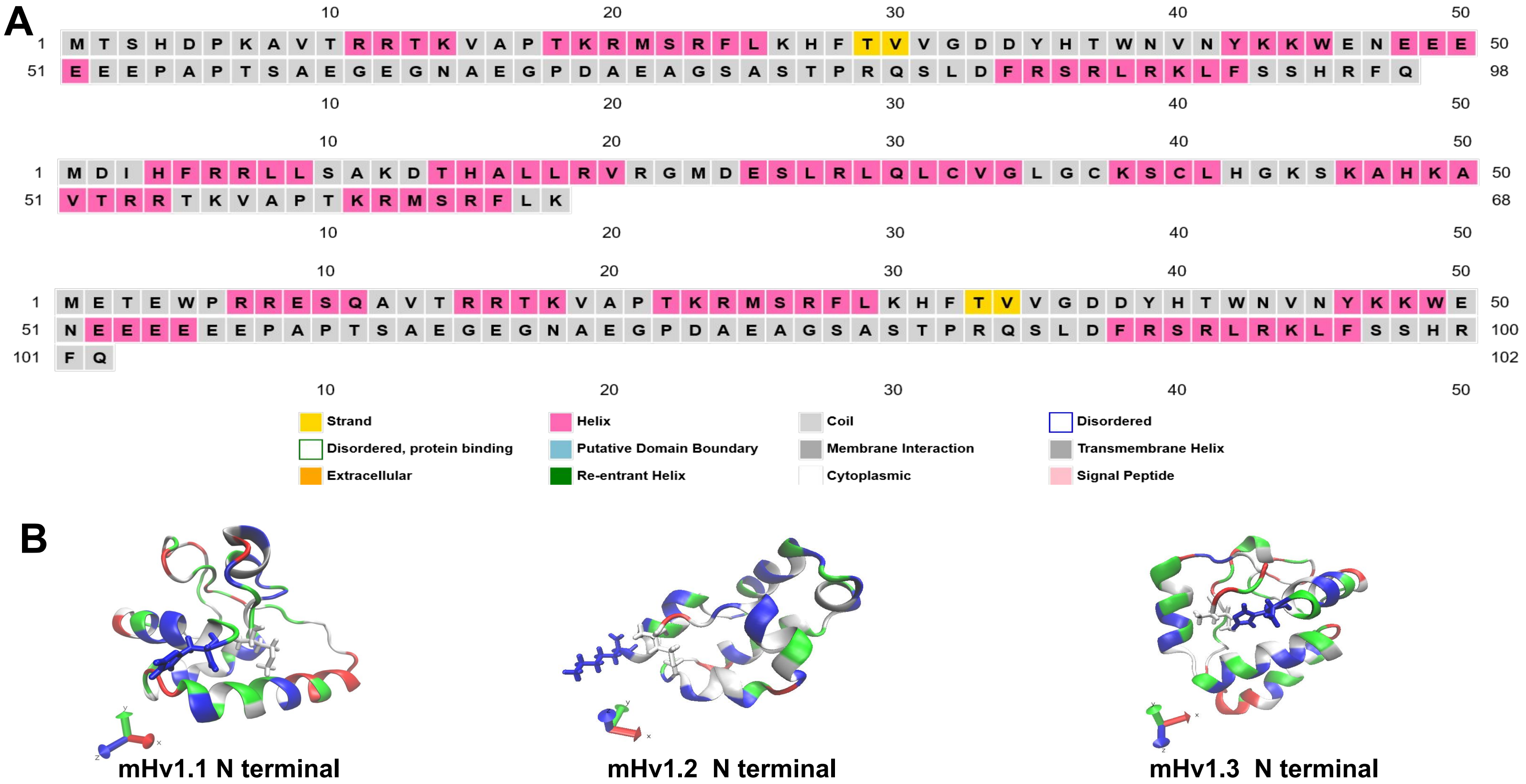

Supplementary Figure 6. Secondary structure and tertiary prediction of the beginning of different N terminal beginning for isoforms of mHv1. (A) PSI-PRED prediction results for secondary structure of mHv1.1 (top), mHv1.2 (middle), and mHv1.3 (pre-bottom). (bottom) Symbology of colors for the prediction. It is observed among all mHv1 channels a high degree of helix motifs and random coils, in proportion and quantity, over the N terminal. **(B)** Ab initio *de novo* predictions of structural models for the N terminal beginning domains of mHv1.1 (left), mHv1.2 (middle) and mHv1.3 (right), with a RMSD of 7.784 A, 6.803 A and 8.087 Å, respectively. Licorice representation shows the N terminal beginning in all isoforms (White Methionine) and the end (Blue Positive charged residue).
